# Transcriptome Analysis of CD4+ T Cells in Coeliac Disease Reveals Imprint of BACH2 and IFNγ Regulation

**DOI:** 10.1371/journal.pone.0140049

**Published:** 2015-10-07

**Authors:** Emma M. Quinn, Ciara Coleman, Ben Molloy, Patricia Dominguez Castro, Paul Cormican, Valerie Trimble, Nasir Mahmud, Ross McManus

**Affiliations:** 1 Department of Clinical Medicine, Trinity College Dublin, Trinity Centre, St James’s Hospital, Dublin, 8, Ireland; 2 Animal and Bioscience Research Department, Grange Research Centre, Teagasc, Dunsany, Ireland; Semmelweis University, HUNGARY

## Abstract

Genetic studies have to date identified 43 genome wide significant coeliac disease susceptibility (CD) loci comprising over 70 candidate genes. However, how altered regulation of such disease associated genes contributes to CD pathogenesis remains to be elucidated. Recently there has been considerable emphasis on characterising cell type specific and stimulus dependent genetic variants. Therefore in this study we used RNA sequencing to profile over 70 transcriptomes of CD4+ T cells, a cell type crucial for CD pathogenesis, in both stimulated and resting samples from individuals with CD and unaffected controls. We identified extensive transcriptional changes across all conditions, with the previously established CD gene IFNy the most strongly up-regulated gene (log2 fold change 4.6; P_adjusted_ = 2.40x10^-11^) in CD4+ T cells from CD patients compared to controls. We show a significant correlation of differentially expressed genes with genetic studies of the disease to date (P_adjusted_ = 0.002), and 21 CD candidate susceptibility genes are differentially expressed under one or more of the conditions used in this study. Pathway analysis revealed significant enrichment of immune related processes. Co-expression network analysis identified several modules of coordinately expressed CD genes. Two modules were particularly highly enriched for differentially expressed genes (P<2.2x10^-16^) and highlighted IFNy and the genetically associated transcription factor BACH2 which showed significantly reduced expression in coeliac samples (log2FC -1.75; P_adjusted_ = 3.6x10^-3^) as key regulatory genes in CD. Genes regulated by BACH2 were very significantly over-represented among our differentially expressed genes (P<2.2x10^-16^) indicating that reduced expression of this master regulator of T cell differentiation promotes a pro-inflammatory response and strongly corroborates genetic evidence that BACH2 plays an important role in CD pathogenesis.

## Introduction

Coeliac Disease (CD) is defined as a common, chronic inflammatory disease of the small intestine that occurs in genetically predisposed individuals and is triggered by exposure to gluten and similar proteins in related grains[1]. The HLA heterodimers, HLA-DQ2 and HLA-DQ8 are necessary but not sufficient to cause the disease, accounting for up to 40% of the genetic susceptibility to CD. The remaining genetic risk is believed to be distributed among an unknown number of non-HLA genes where each gene contributes only a small effect[2]. To date, genome wide association studies (GWAS) have identified 42 non-HLA CD risk loci harboring in excess of 70 candidate genes[3–7].

The majority of genetic variants associated with inflammatory diseases, as revealed by GWAS, are located in non-protein coding sequences and are thought to exert their affect by altering the expression of disease associated genes, many of which remain to be definitively identified[8, 9]. Genetic fine mapping allied to functional studies, including epigenomic analysis and gene expression and eQTL analysis in relevant cell types, will aid in the conclusive identification of the actual disease associated genes and causal genetic variants. Analysing gene expression profiles of patients may allow us to identify dysregulated gene expression and thus molecular pathways with altered activity in disease. Furthermore, by cross referencing with data from gene mapping studies, it may help pinpoint the most likely disease associated genes and genetic variants in the context of specific cells and activation statuses. Such information could potentially be used to refine disease prognosis and risk prediction and may be useful in monitoring disease status[10–12].

Previous genome wide expression patterns in CD have profiled whole duodenal and jejunal[13, 14] biopsy samples and epithelial cells purified from duodenal biopsies[15] using cDNA microarrays. In this study, we specifically profile global CD4+ T cells from peripheral blood to identify gene expression changes in a critical T cell subset known to be pivotally involved in disease pathogenesis[16, 17]. Presentation of deamidated gluten peptides to naïve CD4+ T cells in individuals with CD, leads to T cell activation and up-regulation of a Th1 type immunological response dominated by the production of IFNγ and IL–21[18]. This ultimately leads to the activation of cytotoxic intraepithelial lymphocytes (IELs) which cause much of the intestinal damage by directly killing mucosal epithelial cells.

Therefore, the principle objectives of this study were to assay the transcriptome of CD4+ T cells in CD individuals and those without CD to determine characteristic gene expression patterns in disease, and secondly, to combine this information with that on CD associated variants to establish which genetic associations are mirrored by altered gene expression in a physiologically relevant cell model. To do this, we characterized in a case-control study, the transcriptomes of unstimulated cells and cells stimulated using two different methods, with the aim of maximising our ability to detect genetically determined alterations to gene expression, many of which are only observed following stimulation[9]. Thus CD4+ T cells were stimulated using the PKC/MAP kinase activating agent phorbal myristate acetate (PMA) and the more T cell specific stimulus of anti-CD3/CD28 antibody binding causing activation via the T cell receptor. In contrast to earlier studies, we used RNA-seq to characterize the transcriptome. RNA-seq has become a powerful alternative to microarrays and is considered a more robust and sensitive methodology[19–21], not constrained by limitations affecting microarrays such as dependence on existing knowledge of gene expression and spurious cross hybridization among probe sets[22, 23]. Therefore this represents a departure from previous studies in using RNA-seq in a specific immune cell subset to provide a more comprehensive and refined catalogue of the transcriptome of CD disease relevant cells in a case control study design.

We observe clear differences in the transcriptome between control and CD CD4+ T cells across all three treatments but this difference in gene expression is most highly pronounced following cell stimulation. Genes previously identified as susceptibility factors in CD feature strongly among differentially expressed genes, amongst which, unsurprisingly, there was a strong representation of genes which function in the immune system. Pathway analysis shows enrichment of particularly immune, but also metabolic pathways, indicating a number of molecular systems that appear to be perturbed in CD. We also investigated whether there were identifiable gene networks which were evident in the transcriptomes of patients using weighted gene co-expression network analysis (WGCNA)[24]. This identified *IFNG* and *BACH2* as two important CD ‘hub’ genes emphasizing their interaction with previously identified CD genes, further confirming established[4, 25] and emerging[26, 27] roles for these proteins in CD pathogenesis. In particular we show for the first time a strong signature of BACH2 regulated genes differentially expressed in CD. This implies that this genetically associated transcription factor, which is a master regulator of T cell fate promoting regulatory T cell development at the expense of effector T cells, may be pivotal in the development of this and other inherited immune-mediated disorders.

## Results

We sequenced the CD4+ T cell transcriptomes of 15 individuals with coeliac disease and 11 controls under three different conditions; cells were unstimulated (UNS), stimulated with anti-CD3/CD28 antibody binding and stimulated with phorbal 12-myristate 13-acetate (PMA), comprising a total of 74 transcriptomes. Samples are described in Table 1.

**Table 1:** Sample information for all coeliac individuals and controls sequenced in this study.

Characteristic		Median or n	%^†^	Range			
	**Coeliac individuals**				**anti-CD3/CD28**	**PMA**	**Unstimulated**
**Age**, n 15		57.0		22–75	15	14	13
**Females/Males**, n 15		11/4	73.3/26.7				
**Diagnosis age**, n 15		39.0		0.5–70			
**Diagnosis by age range**, n 15	< 18 years	5	33.3				
	≥18–35 years	2	13.3				
	>35–55 years	5	33.3				
	>55	3	20.0				
**Clinical presentation at diagnosis** *, n 12	Classical CD	9	75.0				
	Non-classical CD	2	16.7				
	Subclinical CD	1	8.3				
**Family history of coeliac disease**, n 15	Yes	5	33.3				
	No	10	66.7				
**Family history of other autoimmune disease**, n 15	Yes	5	33.3				
	No	10	66.7				
**Presence of other autoimmune diseases**, n 15	Yes	4	26.7				
	No	11	73.3				
	**Control individuals**						
**Age**, n 11		50.0		40–70	11	11	10
**Females/Males**, n 11		7/4	63.6/36.3				

* Clinical presentation at diagnosis was classified according to the Oslo definition of CD into; Classical, Non-classical and Subclinical

^†^ Percentages provided are valid percentages

### Power analysis of transcriptome data

It has been previously shown that with a sequencing depth of 10 million reads, approximately 90% of all genes will be covered by at least 10 reads[28]. Furthermore, 30–40 million reads is sufficient to allow technically precise measurement of gene expression for most genes surveyed. As stated above, across the 74 samples we generated 50 ± 11 (mean ± SD) million reads per sample. Using an RNASeq power calculator[29], we estimated that, in a sample size of 11 controls versus 15 cases at average transcript coverage of 10 read counts, we had 93% power to detect a 2 fold change in expression. This increases to over 97% at 20x coverage (S1 Fig) and approaches 100% for more highly expressed genes, indicating that our study was adequately powered to reliably detect changes in gene expression.

**Fig 1:**
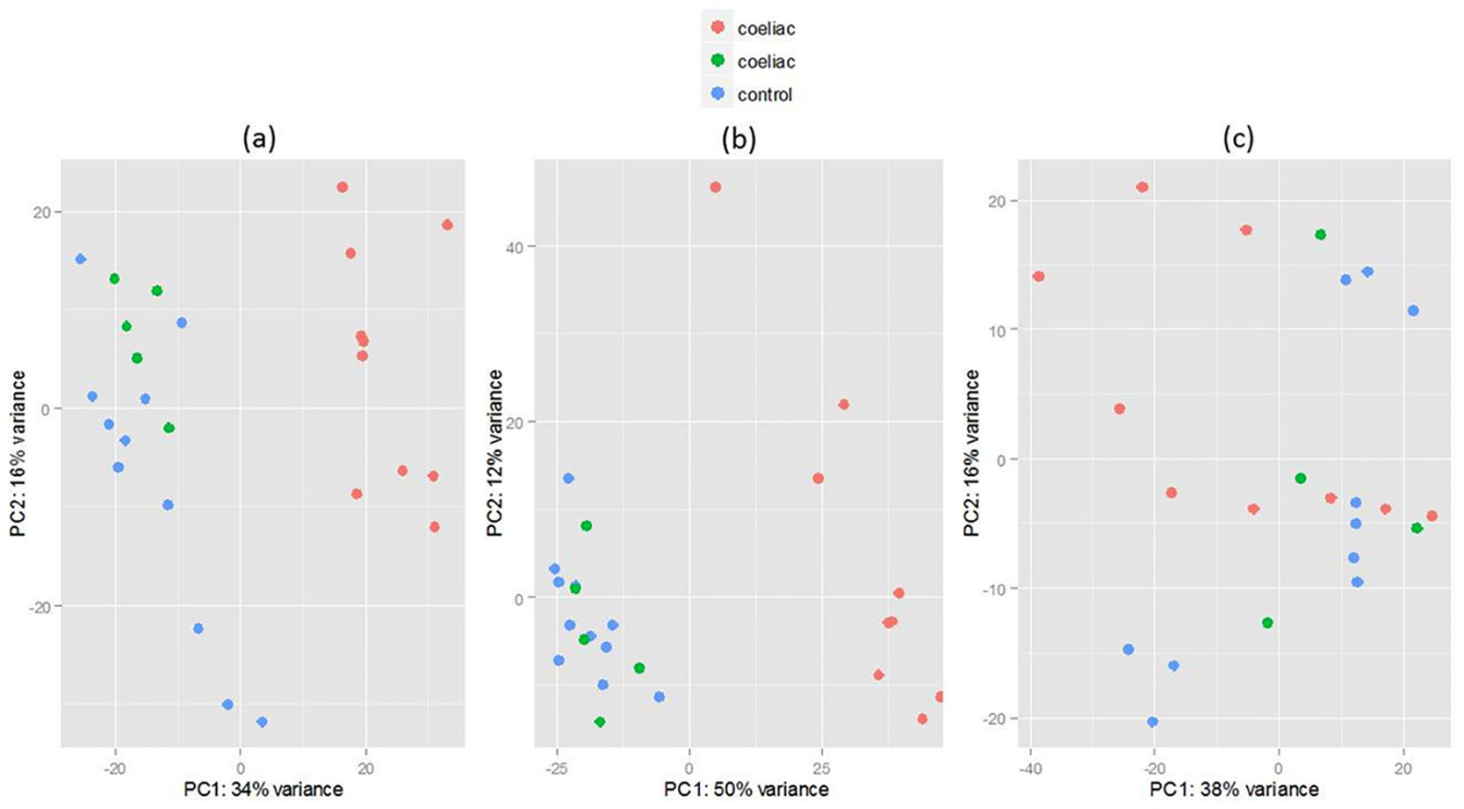
Plots of principal component 1 against principal component 2 based on normalised read counts from the RNA seq datasets. There is distinct separation of the majority of celiac samples and controls in both anti-CD3/CD28 (a) and PMA (b) sample sets however UNS (c) do not form distinct groups.

PCA analysis (Fig 1) of the normalised expression data showed that the unstimulated samples do not form any distinct groups indicating that overall gene expression patterns do not differ between case and control groups in the absence of stimulation. However, samples do cluster when the T cells were activated either by using anti-CD3/CD28 or PMA stimulation (Fig 1a and 1b). Interestingly, the patients separate into two groups with the same 5 coeliac samples (coloured green in Fig 1) clustering with controls upon stimulation with either anti-CD3/CD28 or PMA. Assessment of patient characteristics, including age at sampling, gender, clinical presentation and potential technical confounders such as sequencing batch did not discriminate between the groups (S1 Table). However a higher proportion of patients with control-like expression profiles were diagnosed during infancy (3/5 vs 2/10; median age in years: 2, 0.5–45.0; 44.5, 2–70 P = 0.057). Although all patients self-reported adherence to the gluten free diet at time of sampling, patients with a coeliac like expression profile for whom data was available had higher median tTG levels at the most recent time of sampling (within 6 months) (5.35 U/ml, 0.2–101; vs 0.8, 0.4–3.7; p = 0.098) and a majority of these patients had one or more instances of abnormal tTG (>7.0 U/ml) levels whereas the control-like CD patients had consistently low tTG levels over the previous 3 years on review. Other risk factors appeared more prevalent in the CD-like group, including family history of CD and comorbidity with another autoimmune disease, however these were not significant. As these patients met the patient criteria they were included in the analysis under the speculation that they form part of a subgroup of the disease phenotype or their expression profiles were modified by long term remission.

### Differential expression analysis

**Fig 2:**
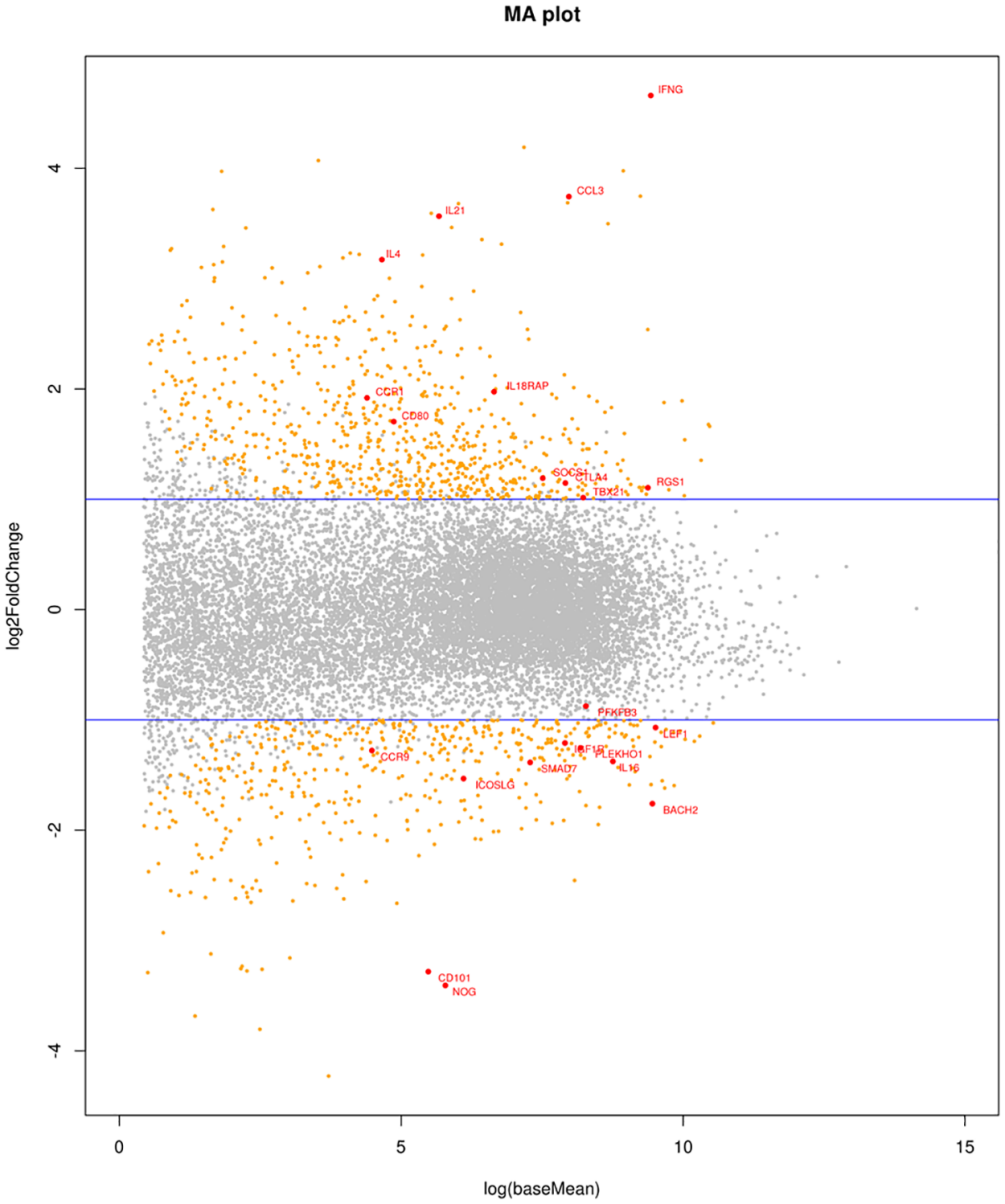
MA plot. Scatter plot of fold-change in expression in cases versus controls (y-axis) against expression level (x-axis). 2 fold up or down regulated genes with adjusted P <0.05 are highlighted in yellow. Differentially expressed genes of interest are highlighted with red dots and labels. Horizontal blue lines indicate 2-fold changes in expression.

Using DESeq2[30], we identified 1069 genes as differentially expressed (DE) between coeliacs and controls in the anti-CD3/CD28 stimulated group (defined as a fold change >2 & FDR of 5%), of which 657 were up-regulated and 412 were down-regulated (MA plot Fig 2 below). The most strongly up-regulated gene in CD samples was *IFNG* (encoding IFNγ) which is known to be highly expressed in the coeliac lesion, with a log2 fold change (log2FC) of 4.6 (P_adj_ = 2.40x10^-11^) and the strongest gene down regulated in CD was *NOG* (Noggin) which functions in the TGFβ signaling pathway specifically inhibiting BMP, which is thought to play a role in gut epithelial cell homeostasis [31] (log2FC = -3.4, P_adj_ = 7.87x10^-08^). Differentially regulated genes in the anti-CD3/CD28 group are listed in S2 Table.

In the PMA group, there were 2,007 DE genes, 1726 being up-regulated and 281 down-regulated. The most strongly up-regulated gene in CD samples was the purinergic receptor *P2RX7* (log2FC = 3.44; P_adj_ = 8.15x10^-10^). P2RX7 has been shown to be heavily involved in the regulation of inflammation in the gut and increased *P2RX7* mRNA levels have been observed in biopsy tissue from individuals with IBD[32]. The most down-regulated gene in CD was the RASD family member 2 (*RASD2*) gene (log2FC = -2.8; P_adj_ = 1.7x10^-05^). RASD2 is a GTP binding protein involved in the mTOR signaling pathway[33]. S3 Table lists all differentially regulated genes in the PMA group.

In the unstimulated (UNS) group, 87 genes were differentially expressed of which 63 were up-regulated and 24 down-regulated (see S4 Table). The most strongly up-regulated gene in coeliac samples was *GRAMD1C* with a log2FC of 2.41 (P_adj_ = 8.45x10^-05^). The most strongly down regulated gene in coeliacs was the RAS-like family 10 member B (*RASL10B*; log2FC = -2.63; P_adj_ = 7.43x10^-06^) neither of which have well documented functions.

**Fig 3:**
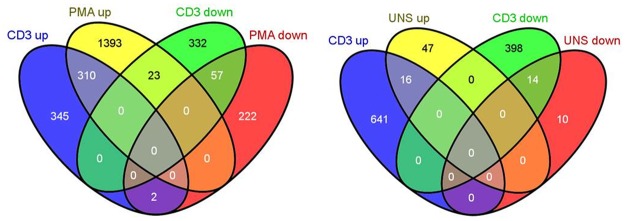
Venn Diagram indicating the overlap in significantly differentially expressed genes in each group. The diagram on the left is a comparison of anti-CD3/CD28 and PMA DE genes while on the right anti-CD3/CD28 and UNS DE genes are compared.

Fig 3 displays the overlap in significantly DE genes (up or down regulated) in each group. The three groups are replicates of the same samples using different or no stimulation. There was high concordance (94% PMA, 100% UNS) in direction of fold change (up or down) between any genes that overlapped under the different stimulations adding confidence that the observed changes in gene expression are real and reproducible.

### Pathway analysis of differentially expressed genes

We conducted pathway enrichment analyses for DE genes in each of the three groups using KEGG pathway annotations.

#### CD3/CD28 Stimulation

There was a strong representation of genes that play a role in the immune system amongst the DE genes; Gene ontology analysis of the anti-CD3/CD28 DE genes using GOseq showed the data to be highly enriched for the GO terms: “Immune system processes”; GO:0002376: (P_adj_ = 1.67x10^-23^) and the “Immune response”; GO:0006955 (P_adj_ = 9.28x10^-18^).

**Fig 4:**
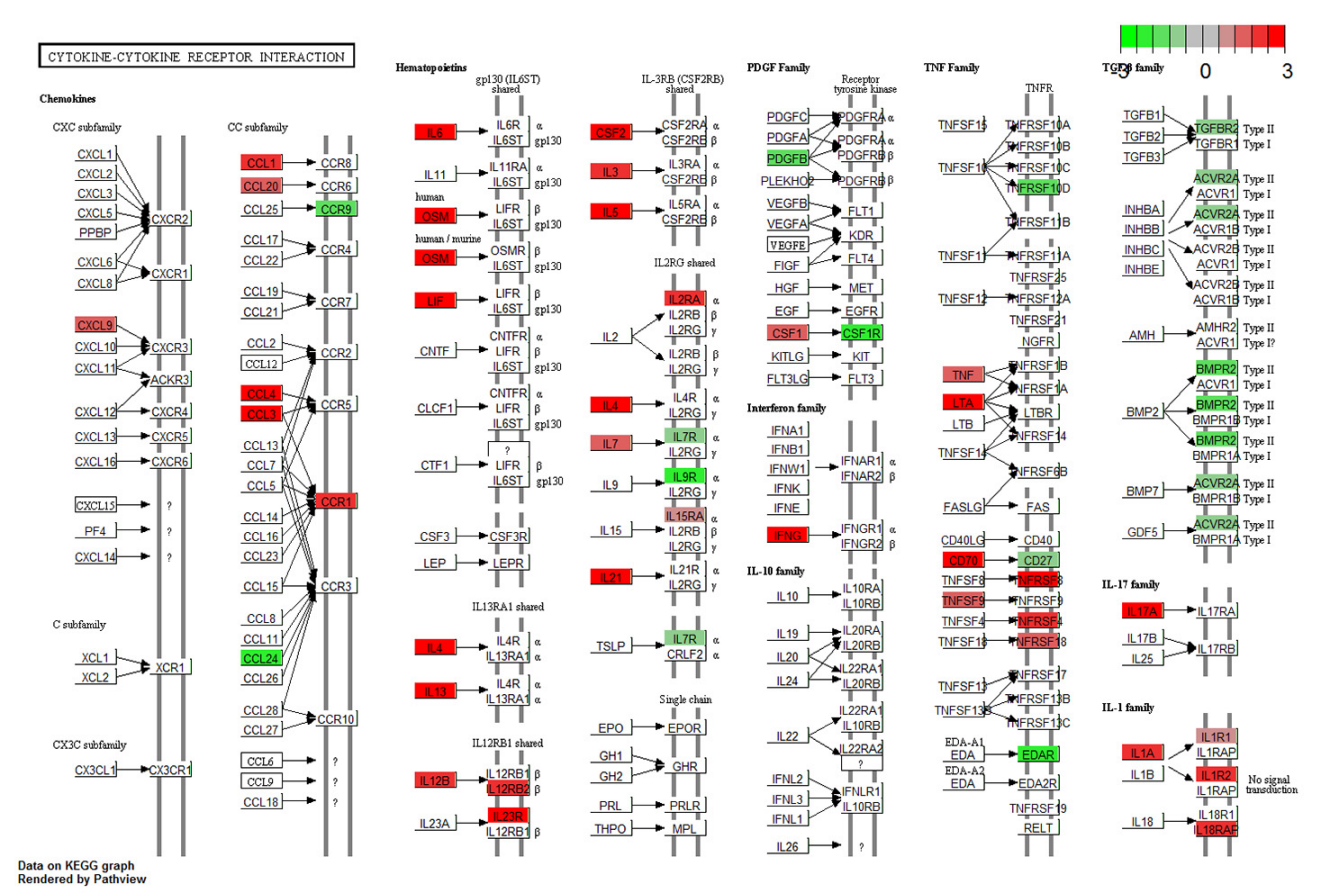
The Cytokine-cytokine receptor pathway. Pathway analysis of genes differentially expressed in the anti-CD3/CD28 stimulated dataset found the Cytokine-cytokine receptor pathway to be most significantly enriched. The direction of fold change in coeliac over controls for the genes involved is indicated in red (up-regulated) or green (down-regulated).

The pathway analysis tool GoSeq[34] identified a number of KEGG pathways enriched by significantly DE genes in the anti-CD3/CD28 stimulated samples. The most significantly enriched pathway was ‘Cytokine-cytokine receptor interaction’. Forty eight of our DE genes belong to this pathway, including 4 genes that have been genetically linked to coeliac disease: *CCR1*, *IL18RAP*, *IL21* and *IL2RA*[4, 7]. A summary of other significantly enriched interesting pathways are listed in Table 2. Pathways enriched for genetically associated genes that show evidence of differential expression in our dataset include: the JAK/STAT signaling pathway, type–1 diabetes, rheumatoid arthritis, chemokine signaling and T cell receptor signaling pathways. The R tool Pathview[35] was used to generate a graphical representation of the cytokine-cytokine receptor interaction pathway as an example, indicating which genes are differentially regulated (Fig 4).

**Table 2:** Pathways enriched for differentially expressed genes. Pathways identified by GOSeq as significantly enriched for genes differentially expressed in anti-CD3/CD28 stimulated samples.

Pathway	#DE genes	# Genes in pathway	Adjusted P value
Cytokine-cytokine receptor interaction	48	259	2.16E-17
Jak-STAT signaling pathway	27	151	1.66E-09
Osteoclast differentiation	21	128	1.36E-06
Metabolic pathways	82	1104	5.37E-05
Toll-like receptor signaling pathway	15	101	7.41E-05
Type I diabetes mellitus	9	43	1.10E-04
Allograft rejection	8	37	1.73E-04
Rheumatoid arthritis	13	89	1.89E-04
Intestinal immune network for IgA production	8	48	1.07E-03
Asthma	6	30	1.09E-03
Fc epsilon RI signaling pathway	11	79	1.24E-03
Wnt signaling pathway	17	149	1.36E-03
Galactose metabolism	6	27	1.74E-03
Graft-versus-host disease	7	41	2.18E-03
TGF-beta signaling pathway	11	84	3.15E-03
Type II diabetes mellitus	7	47	8.56E-03
Chemokine signaling pathway	16	188	1.35E-02
Folate biosynthesis	3	11	1.39E-02
Autoimmune thyroid disease	6	52	1.83E-02
MAPK signaling pathway	21	267	2.36E-02
T cell receptor signaling pathway	10	107	3.97E-02

#### PMA Stimulation

Again there was significant enrichment of immune related genes amongst our PMA stimulated samples (“Immune system processes”; GO: 0002376(P_adj_ = 2.92x10^-16^), “Immune response”; GO: 0006955 (P_adj_ = 1.63x10^-15^).

There was a large overlap between pathways which were significantly enriched for DE genes in the anti-CD3/CD28 and PMA groups, which is unsurprising given the high degree of overlap in the two stimulated DE gene sets. Pathways with excess representation of DE genes after PMA stimulation are shown in S5 Table.

#### Unstimulated

No GO terms or KEGG pathways showed any significant enrichment for genes differentially expressed in the unstimulated group.

### Differential exon analysis

We also tested for differential usage of exons between coeliac and controls using the Bioconductor tool DEXSeq[36]. We identified 647 exons from 542 genes with differential usage between the coeliacs and controls in the anti-CD3/CD28 stimulated samples (FDR<5%). Only 26 of these genes were DE based on the gene level analysis. Interestingly, 4 coeliac associated genes; *YDJC*, *SH2B3*, *ITGA4*, *UBE2E3* were amongst those containing significantly differentially expressed exons but were not DE at the gene level (Table 3).

**Table 3:** Differential exon usage. DEXSeq found evidence of differential exon usage in our anti-CD3/CD28 stimulated samples at 4 genes that have been genetically associated with Coeliac disease.

Chromosome	Exon start	Exon end	Symbol	pvalue	padj	Exon #	log2foldchange
2	182322131	182322154	ITGA4	1.91E-04	0.045	E005	-2.465
12	111843752	111844081	SH2B3	1.08E-03	0.086	E001	-2.869
12	111855923	111856681	SH2B3	8.16E-05	0.034	E003	-1.398
2	181845333	181845533	UBE2E3	2.59E-04	0.050	E008	-1.729
22	21983299	21983476	YDJC	2.19E-04	0.047	E005	-1.196
22	21983597	21983696	YDJC	2.26E-07	0.003	E007	-0.403

### Relating differentially expressed genes to disease association

0ver 40 non-HLA genomic loci have been associated with CD in recent years [4, 37]. eQTL and expression studies[4, 38] have highlighted expression differences at a number of these loci in PBMCs, biopsies[6] and epithelial cells. Therefore we examined if any of these genes were amongst those DE in our anti-CD3/CD28 T cell data. Furthermore, many of the associated loci are large, encoding more than one gene, so we examined whether the expression data could identify a particular gene at a given locus amongst other potential candidates.

The non-HLA loci incorporate approximately 70 protein coding genes. Of these, 11 are significantly DE between coeliacs and controls in our anti-CD3/CD28 stimulated samples, 11 were DE between coeliacs and controls in the PMA stimulated group and 3 were DE in the UNS samples (Table 4). Several show differential and concordant expression under more than one condition, for example *BACH2*, *CCR1*, *SOCS1* and *IL18RAP*, and in total, 21 CD candidate susceptibility genes are DE under one or more of the conditions used in this study. These are tabulated in Table 4 which also shows expression patterns in multi-gene susceptibility loci.

**Table 4:** This table indicates the DESeq2 results across all groups for genes that have been genetically associated with Coeliac disease are also differentially expressed in our data. The direction of fold change in coeliac over controls for the genes involved is indicated in bold (up-regulated) or italic (down-regulated).

Coeliac locus	Gene	CD3+ Log2FC	padj CD3	PMA Log2FC	padj PMA	UNS Log2FC	padj UNS	Candidate genes
1q31.2	RGS1	**1.105**	**2.64E-02**	**1.065**	**6.00E-02**	-0.020	9.88E-01	**RGS1**
2p14	PLEK	0.898	1.50E-01	**1.485**	**1.74E-02**	1.551	1.13E-01	**PLEK**, FBX048
2q12.1	IL1RL1	**2.396**	**2.06E-04**	0.530	4.58E-01	1.769	NA	IL1RL2, **IL1RL1**, IL18RAP, IL18R1
2q12.1	IL18R1	0.920	8.64E-03	**1.859**	**8.93E-05**	0.575	3.26E-01	IL1RL2, IL1RL1, IL18RAP, **IL18R1**
2q12.1	IL18RAP	**1.974**	**2.05E-06**	**1.372**	**1.42E-02**	**1.582**	**3.02E-02**	IL1RL2, IL1RL1, **IL18RAP**, IL18R1
2q33.2	CTLA4	**1.147**	**1.36E-03**	0.201	7.19E-01	0.276	7.25E-01	**CTLA4**, CD28, ICOS
3p21.31	CCR9	*-1*.*277*	*4*.*53E-02*	-0.245	7.95E-01	0.570	5.36E-01	CCR3, CCRL2, CCR2, CCR1, CCR5, *CCR9*, LTF
3p21.31	CCR1	**1.919**	**1.59E-03**	**1.775**	**6.55E-04**	0.726	5.27E-01	CCR3, CCRL2, CCR2, **CCR1**, CCR5, CCR9, LTF
3p21.31	CCR2	0.309	6.84E-01	**1.697**	**2.12E-02**	0.439	6.88E-01	CCR3, CCRL2, **CCR2**, CCR1, CCR5, CCR9, LTF
3p14.1	FRMD4B	**2.012**	**4.40E-05**	0.784	1.95E-01	0.610	3.21E-01	**FRMD4B**
3q13.33	CD80	**1.704**	**7.58E-05**	0.931	1.01E-01	0.227	8.56E-01	**CD80**, ARHGAP31, POGLUT1
4q27	IL2	0.927	6.26E-02	**1.686**	**4.24E-02**	-1.447	1.10E-01	ADAD1, IL21, KIAA1109, **IL2**
4q27	IL21	**3.565**	**1.67E-08**	4.097	NA	1.999	7.44E-02	ADAD1, **IL21**, KIAA1109, IL2
6p25.3	IRF4	0.167	7.62E-01	**1.672**	**6.84E-03**	0.176	8.17E-01	**IRF4**
6q15	BACH2	*-1*.*759*	*3*.*66E-03*	*-1*.*263*	*1*.*72E-02*	-0.722	5.19E-02	*BACH2*, MAP3K7
6q22.33	PTPRK	0.261	6.88E-01	0.649	3.47E-01	*-1*.*876*	*8*.*45E-05*	THEMIS, *PTPRK*
6q25.3	TAGAP	0.237	6.56E-01	**1.155**	**8.58E-03**	0.786	1.59E-01	**TAGAP**
10p15.1	PFKFB3	-0.874	3.60E-04	-0.492	1.25E-01	*-1*.*146*	*1*.*15E-02*	DKFZP667F0711, *PFKFB3*, PRKCQ
11q24.3	ETS1	-0.483	7.60E-02	**1.205**	**1.35E-02**	0.310	5.76E-01	**ETS1**
16p13.13	SOCS1	**1.191**	**8.29E-04**	**1.578**	**1.18E-02**	0.288	7.79E-01	**SOCS1**, PRM1, PRM2
21q22.3	ICOSLG	*-1*.*532*	*1*.*15E-05*	-0.772	8.42E-02	-0.706	2.69E-01	*ICOSLG*

This brings to a total of 25 the genes which have been associated in genome studies and which are either differentially regulated or show alternative exon usage in CD4+ T cells under the conditions utilised in this study.

#### Differentially expressed genes are enriched for coeliac association signals

**Fig 5:**
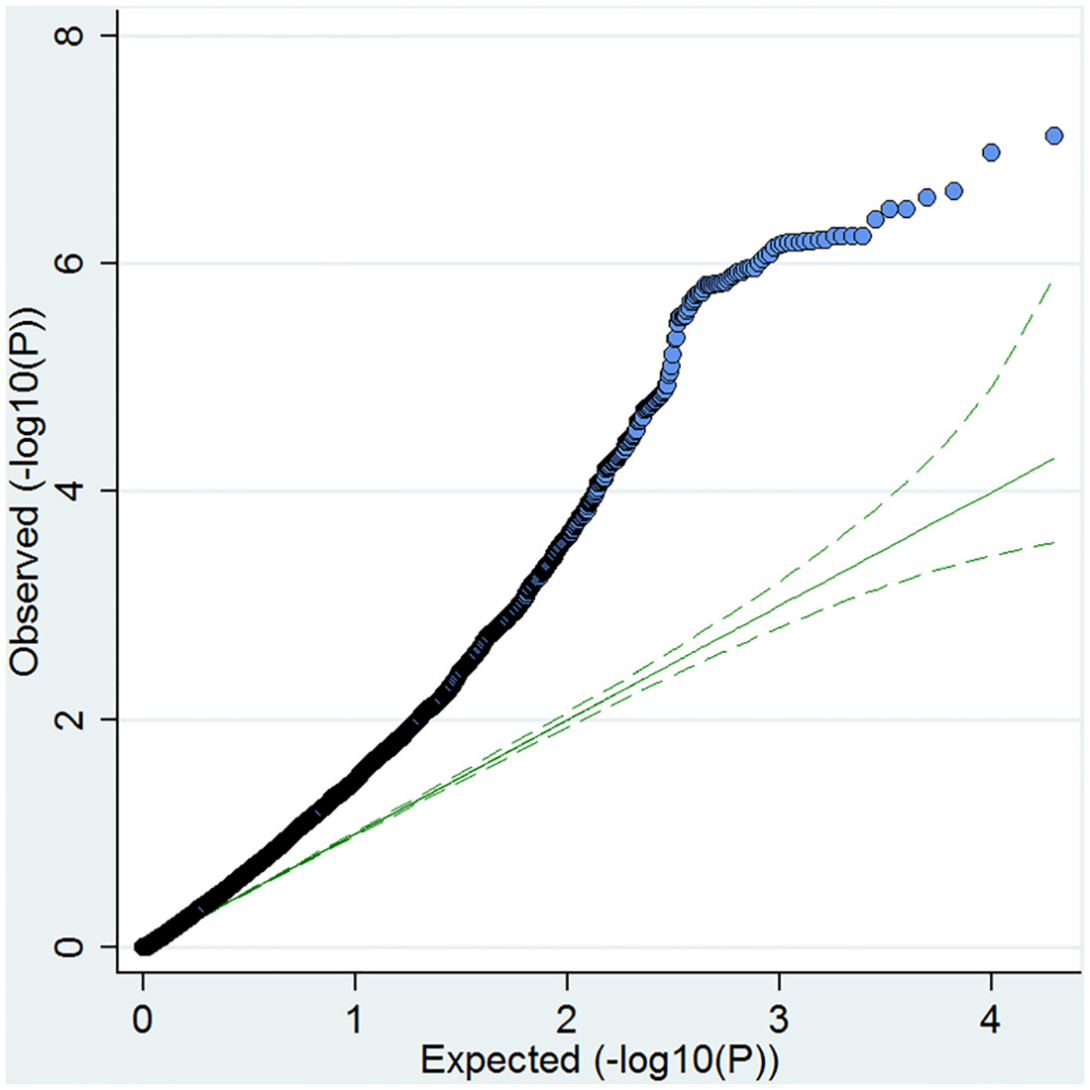
QQ plot of the Immunochip association signals within genes that were differentially expressed between our anti-CD3/CD28 stimulated coeliac and control samples. The expected−log10 p values under the null hypothesis are represented on the x axis whilst the observed values are represented on the y axis.

A QQ plot of SNPs located within the boundaries of DE genes indicated that our gene list was enriched for SNPs that had been associated with coeliac disease using the Immunochip (Fig 5). We used the tool INRICH[39] (Interval-based Enrichment Analysis Tool for Genome-Wide Association Studies) to quantify this finding.

After gene-set-based association analysis and multiple testing correction, there was evidence of strong enrichment for CD associated SNPs amongst our DE genes in the anti-CD3/CD28 group (P_adj_ = 0.002). Interestingly, when up or down-regulated genes were tested for association separately, there was more significant enrichment for up-regulated (corrected P up = 0.01), compared to the down-regulated group (P_adj_ down = 0.07). There was no evidence for enrichment in either the PMA (P_adj_ = 0.57) or UNS (P_adj_ = 0.38) DE gene sets.

We also tested whether any DE genes were found in loci falling below the threshold for GWS in CD. Using INRICH we found that the enrichment was still significant in the anti-CD3/CD28 group (P_adj_ = 0.01) after removal of GWS SNPs. This indicates that SNPs falling short of current benchmarks for GWS are nonetheless associated with differential gene expression in CD patients and could thus be contributing to disease.

### Gene co-expression network analysis

Weighted gene co-expression analysis (WGCNA)[24] can be used to group genes into correlated biologically related expression modules comprised of potentially functionally related genes. Strongly correlated expression levels could imply common regulatory mechanisms, participation in similar biological processes or regulation by a common set of transcription factors. We used WGCNA to analyse the dataset of anti-CD3/CD28 stimulated coeliac cases and controls, as pathway analysis results and INRICH had indicated it to be a more biologically relevant dataset in terms of CD. WGCNA identified 15 modules (M1-M15) in the dataset (Table 5 and S6 Table).

**Table 5:** WGCNA identified 15 modules in our anti-CD3/CD28 stimulated dataset.

Module	# genes in module	ME trait correlation (r2)	Adjusted p-value	# DE genes	Hub genes
M1	190	-0.013	1.00E+00	10	IFIH1, SP110, MYD88
**M2**	**284**	**0.66**	**1.60E-02**	**21**	**CNPY2, MYL6B, PSMD3**
**M3**	**675**	**0.93**	**3.20E-08**	219	**IFNG, TFRC, IL2RA, EVI5**
M4	416	-0.42	9.60E-01	1	FUNDC2, RPL41, RPL39
**M5**	**283**	**0.68**	**1.12E-02**	34	**JAK2, KDELC2, BLM**
**M6**	**1,163**	**-0.96**	**4.80E-11**	203	**BACH2, ZFP36L1, FBXO34, TGFBR2**
M7	867	-0.4	1.00E+00	24	IL11RA, AHSA2, ZNF862
**M8**	**271**	**0.79**	**3.20E-04**	**68**	**FAM54A, TCF19, PSMC3IP**
M9	879	-0.48	4.80E-01	10	ETS1, AKAP11, PAPOLG
M10	712	-0.55	1.44E-01	5	KIAA1109, USP34, ZNF445
**M11**	**204**	**-0.67**	**1.44E-02**	0	**SLC30A7, ZNF845, LZTFL1**
M12	147	-0.13	1.00E+00	0	ADAT2, PRPF39, C1orf27
M13	869	0.55	1.44E-01	20	UBE2L3, C17orf37, TSFM
M14	1,632	0.46	6.40E-01	23	ACTR2, API5, USP14
M15	2,189	0.13	1.00E+00	50	YDJC, CSK, S1PR4

The expression levels of each module were then summarized by the first principal component (module eigengene) and correlated with disease status. Additionally, each gene in a module was ranked by its connectivity or module membership in order to identify module ‘hub genes’ or genes with the greatest connectivity.

We found significant correlation between 6 modules and CD patient status. Modules 2, 3, 5 and 8 were positively correlated with disease status, meaning that genes in these modules show greater expression in CD. Conversely, modules 6 and 11 were negatively correlated, meaning they comprise genes showing predominantly lesser expression in CD cases. R^2^ and P values for these correlations are listed in Table 5.

Of these modules, M3 and M6 were of greatest interest as both were significantly enriched for differentially expressed genes (P<2.2x10^-16^, Fishers exact) and showed the strongest correlation with disease status.

**Fig 6:**
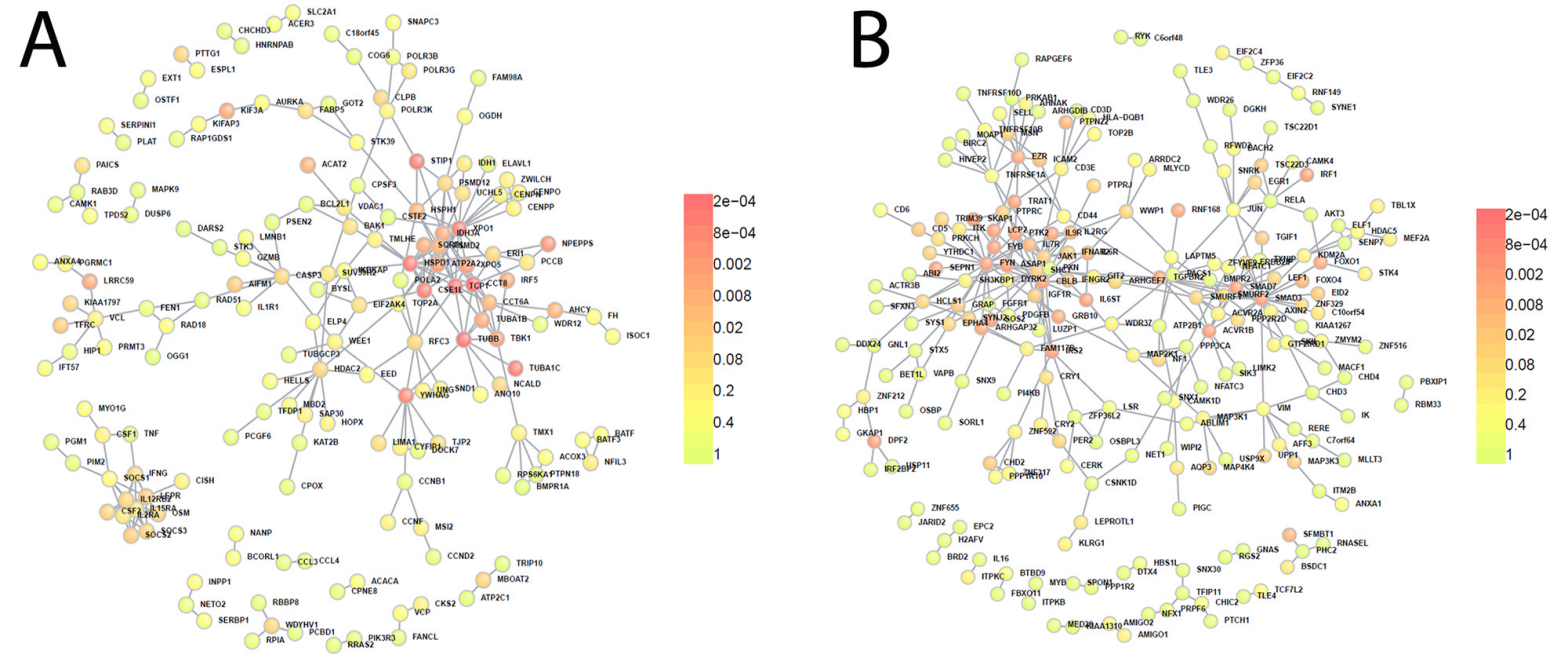
Protein interaction networks for modules 3 and 6. Genes in M3 (a) and M6 (b) showed enrichment for protein-protein interactions. Colour code represents the probability that a protein would be as connected to other proteins by chance based on 1000 permutations.

We used the protein interaction tool DAPPLE[40] to test whether these two modules had more protein-protein interactions than expected by chance. Both M3 (P = 0.0009) and M6 (P = 0.001) were enriched for direct protein connections (see Fig 6a and 6b) which supports the interpretation that the co-expression of genes we observe in the WGCNA analysis is caused by functional interactions of these genes.

The M3 module was strongly associated with coeliac disease status (r^2^ = 0.93, P_adj_ = 3.2x10^-8^) and contained over 200 DE genes. Pathway analysis of this module using MetaCore showed enrichment for the Immune response: T cell subsets (P_adj_ = 1.8x10^-4^), the JAK/STAT Pathway (P_adj_ = 6.3x10^-3^). M3 contains a number of genes genetically associated with CD, including *IL18R1*, *CCR5*, *PTPRK*, *CTLA4*, *RGS1*, *IL18RAP* and *SOCS1* which we show to be differentially expressed. DAPPLE[40] reported a list of 56 genes to prioritise in M3 that have more direct protein connections in the module than would be expected by chance. Interestingly one of these genes is *IFNG* which along with being the most significantly DE gene in M3 is also a hub gene in this module (MM = 0.91, P = 4.15x10^-9^). The immunologically related genes *IL12RA* (MM = 0.95, P = 4.3x10^-11^) and *IL32* (MM = 0.95, P = 4.6x10^-11^) are also hub genes in M3.

The M6 module showed the greatest association to coeliac disease status (r^2^ = -0.96, P_adj_ = 4.8x10^-11^), was significantly enriched for DE genes (P<2.2x10^-16^, Fishers exact) and also showed enrichment for coeliac associated loci when tested using INRICH (P_adj_ = 0.004). Pathway analysis of this module showed enrichment for genes involved in TGFβ receptor signaling (P_adj_ = 6.9x10^-3^) and T cell receptor signaling (P_adj_ = 2.2x10^-2^). Known or putative coeliac susceptibility genes in M6 include *BACH2*, *ZFP36L1*, *SH2B3*, *PFKFB3*, *TNFAIP3*, *ICOSLG* and *FASLG*. Interestingly, the TGFβ receptor 2 *(TGFBR2)* is one of the top hub genes in this module (MM = 0.95, P = 6.38x10^-12^), as is the coeliac associated gene *BACH2* (MM = 0.96, P = 2.8x10^-12^) which is required for TGFβ induced FOXP3 expression and thus normal Treg function[41]. BACH2 was also found to participate more often with other proteins in the interaction network built using DAPPLE[40] than expected by chance.

*BACH2* is a very interesting candidate gene for involvement in coeliac disease. It is a transcription factor which has an important role in balancing tolerance and immunity, and appears to exert a suppressive effect on inflammation[27, 41, 42] by controlling T cell differentiation. We see a significant decrease in *BACH2* expression in both our anti-CD3/CD28 (Log2FC -1.75; P_adj_ = 0.0037), PMA stimulated (Log2FC -1.26; P_adj_ = 0.017), and unstimulated (Log2FC -0.72; P_adj_ = 0.052) coeliac samples, which is concordant with the findings that suggest risk alleles for the disease are associated with *BACH2* down regulation[4].

We investigated if genes previously identified as subject to BACH2 regulation were differentially regulated in our dataset. A recent study in mice by Vahedi et al[43] looked at regions of the genome that act as super enhancers (SE) in CD4+ T cells. They identified a network of BACH2 regulated genes composed primarily of cytokines and cytokine receptors, many of which are critical for T cell function. On examining our RNA Seq data, we found our differentially expressed genes were significantly enriched for BACH2 targets (P<2.2x10^-16^, Fishers exact test). Ninety eight genes regulated by BACH2 were differentially expressed between coeliacs and controls in our dataset. Furthermore, expression patterns of these genes were correlated with a decrease in BACH2 expression in that 23/32 (72%) genes that are apparently induced by BACH2 (WT v KO transcriptome data) are down-regulated in our coeliacs and 60/66 (91%) genes that are apparently repressed by BACH2 are up-regulated. (These genes are listed in S7 Table).

To further assess this finding, we used Gene Set Enrichment Analysis (GSEA) [44] to examine if BACH2 regulated genes in the Vahedi et al [43] data set were enriched among our DE genes across all three conditions (anti-CD3/CD28, PMA and UNS). We also analysed if the changes in gene expression observed by Roychoudhuri et al [27] in mouse CD4SP thymocytes following BACH2 knockout were significantly enriched in these data. In the anti-CD3/CD28 expression data, we observed a very extreme distribution of BACH2 regulated genes as identified by Vahedi et al, with all of these genes found in the upper and lower ends of the spectrum of DE genes (S2 Fig) (normalised enrichment score [NES] = 1.716; FDR q-value = 0.00127). The DE gene set of Roychoudhuri et al was not significantly enriched among our DE genes (NES = -1.226; FDR q-value = 0.224). There was no evidence of enrichment of either gene set among our UNS DE genes (Vahedi FDR q-value = 0.317, NES = 1.249; Roychoudhuri FDR q-value = 0.292, NES = 1.098) or our PMA DE data set (Vahedi FDR q-value = 0.145, NES = 1.305; Roychoudhuri FDR q-value = 0.275, NES = 1.316). The different associations seen in these two gene sets may relate to the fact that the Vahedi data set consists of super enhancer genetic architecture in CD4 T cells and thus at the top of the hierarchy of BACH2 regulated genes, while the Roychoudhuri gene set is composed of BACH2 regulated DE genes which are likely amplified by various downstream regulatory circuits in this cell type (mouse CD4SP thymocytes). (See S8 Table for gene set rank and enrichment scores).

## Discussion

With a known environmental trigger, gluten and the HLA-DQ genes well characterised as genetic risk factors, coeliac disease is one of the best understood of all complex diseases. While the general mechanism, of gluten provoking innate and adaptive immune responses orchestrated by T cells, is well established, much of the complex network of processes that ensues remains to be elucidated—for example, how differences in the regulation of disease associated genes affects molecular circuitry in individual cells.

Recent GWAS and fine mapping studies have identified over 40 CD susceptibility loci. However, it is not always clear which is the true causal gene or genetic variant at a given locus. Associated variants are predominantly found in non-coding DNA and are believed to contribute to disease by modifying the expression of nearby genes often in a cell specific manner[45]; altered gene expression affecting particular molecular pathways is therefore likely to be an important mechanism of disease. In our data, pathways significantly enriched with differentially expressed genes include T cell development, T & B cell co-stimulation, cytokines, chemokines, NK cell activation and interferon-γ production among others.

Clearly, CD is caused by the interaction of several different cell types, including antigen presenting cells, CD4+, CD8+, NK, and epithelial cells. Genomic approaches do not discriminate between cell types and it is unlikely that all genes or SNPs identified in these studies are functional in every cell type. To date, transcriptomic studies in CD have generally used mixed populations of cells (for example whole biopsy material) even though it is certain that each has different transcriptional profiles, potentially relevant to disease activity. Thus, we aimed to profile the transcriptome specifically of CD4+ T cells, which are known to be centrally important in CD pathogenesis[16, 17]. We therefore characterised genome-wide differences in transcription between individuals with coeliac disease and unaffected controls in resting cells and cells activated using two different stimulations (anti-CD3/CD28 and PMA). This is, to our knowledge, the first case control study to apply mRNA sequencing specifically to T cells from coeliac disease patients. We compared the transcriptomes of unstimulated and stimulated T cells, since the latter should more closely model the scenario in the coeliac lesion which is the site of vigorous immune activity. Also, the use of three independent treatments on each sample allows for comparisons of the effect of each treatment and also represents an internal replication under different conditions with the results proving highly concordant.

The data gave us the opportunity to (i) compare gene expression between cases and controls; (ii) specifically assess whether genes that have been genetically associated with the disease were being DE; (iii) and also look for known and novel aspects of pathogenesis in the transcriptome of this specific cellular compartment.

Whether or not the T cells were stimulated had a major impact on our findings. Stimulation using anti-CD3/CD28 and PMA resulted in clustering of our coeliacs and controls which was not obvious in unstimulated cells. Clustering patterns for PMA and anti-CD3/CD28 were highly similar. Thus, the transcriptome of peripheral T cells from the majority of patients is considerably different from that of controls under the conditions employed here, implying that these differences are ultimately genetically determined and relevant in disease pathogenesis. However we also found evidence of a potential subgroup of coeliacs whose mRNA expression (following stimulation) was similar to controls. As discussed in the results section, this could represent a subgroup which differs to the other CD patients in the study in terms of disease processes, genetic susceptibility or possibly adherence to the gluten free diet. The fact that the same individuals reacted similarly to two different types of stimuli argues that this is not an artefactual finding. Interestingly, this kind of transcriptional signature has been observed in expression studies of other autoimmune diseases[46, 47] and in coeliac disease Anderson and colleagues previously reported that CD4+ T cells from approximately one third of patients failed to produce IFNγ in response to gliadin stimulation [48]. A larger study of CD patients or use of different conditions (e.g. different time course following stimulation) may help evaluate these observations.

Anti-CD3/CD28 stimulation is considered more physiologically relevant as it mimics stimulation by antigen-presenting cells; however we observe that it has less extensive effects than PMA, which stimulates cells by activating the PKC and MAP kinase signaling pathways. Using both agents allowed us compare the transcriptome-wide effects of these different stimulations. Genes DE following anti-CD3/CD28 stimulation were enriched for pathways such as dysregulated cytokine and T cell receptor signaling, in keeping with what is known of disease mechanisms. Furthermore, this list of genes was shown to be enriched for coeliac associated loci. The PMA and anti-CD3/CD28 data were highly concordant in terms of direction of fold change (r^2^ = 0.71) regardless of significance which acts to substantiate the findings since the samples were replicas treated independently and differently once split. However almost double the number of genes were DE after treatment with PMA with pronounced enrichment of general cell metabolism pathways (S5 Table). Hence we concluded that the anti-CD3/CD28 data was more representative of the *in vivo* state of immune activation and so focused on this for network analysis.

Although genetic studies of CD have only identified ~70 genes in the disease, we observe large differences in mRNA expression affecting >1000 genes in the anti-CD3/CD28 (and more than 2000 in the PMA) samples. Many genetically linked genes are represented in this group. It is likely that many of the other DE genes are the downstream amplification effects of the dysregulation of key disease associated genes such as transcription factors or signaling molecules, any perturbation of which could have widespread effects on the transcriptome. Most of these effects are small in terms of fold change of expression; however their potential importance to pathogenesis is demonstrated by IFNγ, which displayed a ~25 fold increase in expression in cases compared to controls. This shows that the documented high expression of IFNγ in the coeliac lesion is probably indirectly genetically determined even though *IFNG* itself is unlinked to CD. The subtle differences in expression in other genes may combine to have a more cumulative downstream impact on pathways affecting disease. This can be seen in the enrichment of differentially expressed genes in a number of self-evidently important immune related pathways in our data e.g. the JAK/STAT and TGFβ pathways.

We show that 21 of 70 putative CD susceptibility gene candidates are DE under one or more of the conditions tested here. A further 4 genes show differential exon usage bringing the total to 25. In regions where there is more than one candidate gene, our results distinguish several as the more likely candidate gene in CD4+ T cells. In region 2q12.1 however, three genes, both of the IL18 receptor genes and the IL1 receptor gene *IL1RL1*, all show greater expression. Since several studies show eQTLs in this region acting on both IL18 receptor genes, it may be that they both represent susceptibility genes for CD. eQTLs with overlapping effects on *IL18R1* and *IL1RL1* have also been reported[49] indicating complex regulatory associations. In the chemokine receptor cluster, *CCR1*, *CCR2* and *CCR9* are differentially regulated in this study, the latter being of particular interest since it is involved in gut homing.

Our findings of DE are in good agreement with a recent study by Plaza-Izurieta et al[6], who looked specifically at the expression differences of 45 CD associated genes in whole intestinal biopsy tissue from 15 coeliac cases and controls. They showed 15 of the 45 genes to be differentially expressed. Five of these, *SOCS1*, *CTLA4*, *ZFP36L1*, *ICOS*, and *ICOSLG* were also significantly DE in the same direction in our anti-CD3/CD28 stimulated samples (although *ZFP36L1* and *ICOS* did not pass our FC cutoff i.e. >2) and *PLEK* in our PMA stimulated samples, while the remaining 9 genes were not significantly DE under any of our conditions. Other notable DE genes which are either genetically linked or thought to be functionally involved in the lesion and are DE in our study include *IL21* (Log2FC 3.6, P = 1.7x10^-8^), *IL23R* (2.5, 7.5x10^-7^), *IL15R* (1.01, 6.2x10^-6^).

Common co-morbidities of coeliac disease include type 1 diabetes (T1D), rheumatoid arthritis (RA), ulcerative colitis (UC) and Crohn's disease. There is considerable overlap between loci for coeliac disease and other immune-related diseases and this is reflected in our data, since our DE genes are enriched for pathways relating to several of these diseases (Table 2).

Given this sharing of molecular mechanisms, we investigated whether DE genes failing to achieve genome wide significance (GWS) for CD had been genetically associated with another autoimmune disease at GWS level, and found that ~14% (145 out of the 1069) had been linked to at least 1 or more (S9 Table). Some genes of interest include CD226, a cell surface receptor involved in T cell adhesion and activation (associated with Crohn’s disease, RA, T1D and UC) which is upregulated in our coeliacs and was previously strongly associated with CD, falling however short of GWS (P = 10^−7^)[7]. CTSH (cathepsin H) is also upregulated in our coeliacs and is genetically associated with MS and T1D where it has been linked to β-cell function during disease progression[50]. The CTSH SNP rs34843303 is strongly associated with CD (P = 2.5x10^-6^) and is an eQTL which affects CTSH levels in B cells and monocytes[51]. LONRF2 is down-regulated in coeliac patients and was previously identified as being associated with CD[52], T1D[53] and RA[54].

Of novel interest is *ZPBP2* (log2FC 1.06, P = 0.025) which maps to a locus associated with several other immune mediated diseases (T1D, Primary Biliary Cirrhosis, Crohn’s Disease, UC, RA and asthma) also encoding candidate genes *ORMDL3*, *GSDMB* and *IKZF3*, none of which are differentially expressed in our data; and *CSF2* (encoding GM-CSF; log2FC 2.45 P = 7.5x10^-08^) which has been associated with CD, UC and RA. CSF2 is similarly overexpressed in a recent RNA-seq analysis of psoriatic skin lesions[21].

Network analysis using WGCNA identified several co-expression modules associated with coeliac disease. Two of these modules (M3 and M6) were highly enriched (p<2.2x10^-16^) for DE genes.

The most highly correlated module is M6 (r^2^ = -0.96, P = 4.8x10^-11^), highlighting the CD susceptibility gene *BACH2* as a key hub gene. Polymorphisms at *BACH2* have been associated with numerous inflammatory diseases including Crohn’s disease[55] and Type 1 diabetes[56]. We find that BACH2 mRNA levels are substantially decreased in coeliacs compared with controls, which is in agreement with eQTL studies in peripheral blood showing CD risk alleles at *BACH2* are associated with decreased expression[4].

BACH2 is emerging as a crucial regulator of immune cell differentiation. It is a transcription factor which has been shown to bind super enhancers that control cell identity[43]. In CD4+ T cells, it inhibits the expression of genes needed for the development of effector cells (Th1, -2, -17), and promotes the formation of Treg cells. This immunoregulatory role is essential, and when under expressed, the formation of Tregs is reduced, leading to fatal levels of inflammation in the case of knockout animals[27]. Notably, these animals displayed significant inflammation and immune cell infiltration of the gut. As stated, lesser BACH2 expression is seen in coeliac patient samples under all conditions (anti-CD3/CD28 Log2FC -1.76, P_adj_ = 0.0037). Our differentially expressed genes were significantly enriched for BACH2 super enhancer regulated targets (P<2.2x10^-16^; GSEA FDR q-value = 0.00127) and genes previously shown to be repressed by BACH2 were up-regulated in our coeliac individuals. BACH2 deficient cells produce greater amounts of effector cell related transcripts[27, 41, 42] and cytokines representative of Th1, Th2 and Th17 cells show increased expression in our data, as might be expected if BACH2 represses T effector cell activity or differentiation. However at the level of master cell fate regulators, only TBX21 is DE using our criteria, potentially showing an expansion of Th1 cells in the CD sample. This may be directly attributable to BACH2 since TBX21 shows increased expression in *Bach2* knockout mice[43]. Enrichment of the BACH2 super enhancer regulated genes is only observed in the anti-CD3/CD28 stimulated samples and not in the unstimulated samples, consistent with BACH2 controlling the differentiation of cells from naïve to effector or regulatory states. If replicated in the coeliac lesion, this could plausibly result in an abnormal or exaggerated local immune response. While there is no evidence of reduced Treg populations in CD patients, several reports suggest that they are unable to suppress T effector cells effectively[57–59] a finding also seen in Tregs from *Bach2* KO mice [27]. Interestingly, TGFBR2 which is downregulated in *Bach2* KO mice, is also a hub gene in the M6 module and TGFβ signaling has been shown to require BACH2 to maintain Treg function[41]. Our pathway analysis (see Fig 4) shows receptors of the extended TGFβ/BMP family to be comprehensively down-regulated although not all meet our cutoff for significance. Taken together, these observations support decreased immune suppression via Treg / T effector cell homeostasis as an important mechanism in CD pathogenesis.

M3 is the next most highly correlated module with coeliac disease (r^2^ = 0.93; P = 3.2x10^-8^). IFNγ is a hub gene in M3 and our network analysis showed that a substantial proportion of our DE genes were co-expressed with *IFNG* in CD4+ T cells, indicating that these changes are diagnostic of CD status and may be causatively associated. Our findings are interesting in the context that Kumar et al[26] recently noted that 30% of plausible genetically associated coeliac genes (15/49) showed evidence of co-expression with IFNγ and of these, 7/11 genes for which data is available in Fairfax et al[9] were significantly induced by IFNγ in monocytes.

This is relevant because we show that IFNγ is the most significantly upregulated gene in peripheral CD4+ T cells. IFNγ is very highly expressed in the coeliac lesion where it plays a critical role in disease pathogenesis[60, 61]. It is produced by T cells and powerfully promotes the Th1 response characteristic of CD. IFNγ signaling is via the JAK/STAT pathway, which is linked to a variety of inflammatory diseases and is strongly represented amongst DE genes enriched in our data (P = 1.66x10^-6^). This all goes to illustrate the centrality of IFNγ expression to the network of DE genes despite there being no genetic association of the *IFNG* locus itself and suggests that this is a downstream effect of inherited susceptibility variants which ultimately regulate IFNγ expression.

A plausible cause of this high IFNγ expression is IL18RAP, which is co-expressed with IFNy in M3, and is DE in our data (log2FC 2, P = 2.05x10^-6^). *IL18RAP* (encoding IL18Rβ) has been linked to CD in several genomic studies[4, 37] and combines with IL18Rα (encoded by *IL18R1*) to form the IL18 receptor which is expressed predominantly on T and NK cells. IL18 is a key IFNy stimulating cytokine expressed by monocytes, dendritic cells and epithelial cells of the gastrointestinal tract[62]. Thus upregulation of the IL18 receptor could be expected to lead to the increased IFNγ expression typically seen in the CD lesion. IL18R1 (IL18Rα) is also overexpressed, but to a lesser extent (anti-CD3/CD28 log2FC = 0.92, P_adj_ = 0.086; PMA log2FC = 1.86, P_adj_ 9x10^-5^). IL18 is known to act synergistically with IL12 in inducing IFNy production in Th1 cells, and these interleukins reciprocally upregulate each other’s receptors[63]. This is seen also in our data, with both IL12RB2 (log2FC = 2.13, P_adj_ 7.8x10^-5^) and IL12RB1 (log2FC = 0.38, P_adj_ = 0.03) significantly overexpressed in anti-CD3/CD28 stimulated patient cells. Finally, it is interesting to note that *IL18RAP* (and *IFNG*) appears to be repressed by BACH2 according to Vahedi et al[43] indicating it may be a nexus for CD associated risk factors. Thus the upstream ingredients for increased IFNγ expression are present in our anti-CD3/CD28 stimulated CD4+ T cells.

In accordance with our study, Plaza-Izurieta et al[64] found that IL18RAP was significantly overexpressed in intestinal biopsies of active CD samples. Koskinen et al[65] also showed overexpression of IL18RAP in CD which they suggest may be in part explained by higher expression levels of a newly identified 35kDa alternative IL18RAP isoform. However we see no evidence of differential exon usage within the IL18RAP transcript itself or in exon usage between cases and controls.

However these observations conflict with those of Dubois et al[4], who reported that the IL18RAP proximal coeliac disease associated eQTL (rs917997), correlates impressively with IL18RAP down-regulation (P = 7.4x10^-87^). This correlation was shown in unstimulated peripheral blood leukocytes and the possibility exists that it behaves differently in resting compared to stimulated cells. Indeed, Fairfax et al[9] showed that the majority of eQTLs were active only upon cell stimulation while many constitutive eQTLs in resting cells were not functional post stimulation and a similar observation on SNPs associated with autoimmune diseases was made by Lee et al in dendritic cells[66]. As stated above, lower expression of BACH2 may also contribute to higher IL18RAP expression. Finally, another difference is that we are testing CD derived cells compared to controls, rather than looking for an eQTL effect which this study is insufficiently powered to do. While we highlight IFNy as a hub gene of the M3 module many genes of this module are dysregulated and may have an impact on disease.

There are several limitations to this study. Clinical presentation in CD is highly variable; however this study was not sufficiently powerful to correlate changes in gene expression with patient characteristics, nor was it possible to relate gene expression to genetic profile. Questions such as whether the transcriptomic signatures of CD are specific to individual T cell subpopulations (Th1, Th2, Th17 and regulatory T cells) or profiling gene expression at different time points post activation or in an antigen specific subgroup rather than a peripherally derived global CD4+ T cell population were beyond the scope of this study. This study only considers the coding mRNA from these patients—however non coding RNAs have been implicated in the disease and it would be interesting to also investigate these between patients and controls[67].

That being said, as the majority of expression and eQTL studies in CD have been performed using PBMCs because of the ease of collecting them, this study represents an advance as it examines expression changes in a cell type known to play a strong role in disease development, hence generating more disease relevant information. It is clear that much future functional assessment of the transcriptome needs to be carried out using varied activation protocols in defined cell types.

In conclusion, using an unbiased approach, we characterised over 70 transcriptomes from coeliac patients and controls in both resting and stimulated cells. We identified large transcriptional changes that significantly mirror what has emerged from genetic studies of the disease. Differential expression is strongly associated with genes of immunological function particularly in the anti-CD3/CD28 stimulated group, with PMA stimulated cells showing a larger signature representing metabolic and proliferative changes. Genes which have been associated with CD in genome scans are enriched in our DE genes and our data provides corroborating evidence for several genes which fail to meet genome wide significance levels. Genes shown to be DE in this study are strongly associated with several co-expression networks, the most strongly of which feature IFNG and BACH2, which is genetically associated with CD and down-regulated under all conditions tested. We identify a strong association of CD with a network of BACH2 regulated genes, supporting emerging evidence of an important role of BACH2 in the regulation of T cell differentiation and prevention of autoimmune disease.

## Materials and Methods

### Ethics Statement

Written and informed consent was obtained from all subjects. The study was approved by the St. James’s Hospital, and the Adelaide and Meath and National Children’s Hospital (AMNCH) Research Ethics Committee.

### Collection and processing of samples

Detailed sample information is given in Table 1 and S1 Table. Our control samples were healthy volunteers (not screened for CD) from the general population. None of our volunteers were taking immunosuppressive drugs nor had any known autoimmune disease or family history of any autoimmune disease. All individuals are Caucasian and have Irish ancestry. 30mls of blood was collected from each coeliac patient (mean age 53 +/-12) and control subjects (mean age 51 +/-9) with 24mls used for CD4+ T cell isolation and subsequent RNA extraction.

CD4+ T cells were isolated using positive selection from each blood sample using CD4+ microbeads under standard procedures (Milteny Biotech GmBH). CD4+ T cells were then divided into 3 separate groups and stimulated with either 10ul of immobilized anti-CD3 antibody (1mg/ml) (HIT3a,Becton Dickinson) and 1ul anti-CD28 antibody (1mg/ml) (CD28.2, Becton Dickinson) for 24 hours at 37°C, 10ul (1ng/ml) Phorbal 12-myristate 13-acetate (PMA) for 4 hours at 37°C or left unstimulated (UNS) to represent basal conditions and incubated for 24 hours at 37°C. The purity of our CD4+ T cells was quantified at various stages throughout the isolation process with yields exceeding 98.5% purity post culture (see S1 File).

Total poly(A)^+^ RNA was extracted from ~10^−6^ cells using the Qiagen RNeasy Micro kit according to the manufacturers protocol for all samples. The RNA quality was checked using an Agilent 2100 bioanalyser and only samples with a RIN of 8 or greater were included in the study.

### mRNA Sequencing

cDNA library preparation and sequencing was performed on an Illumina HiSeq™ 2000 using 50 base pair single end reads (GATC Biotech, Konstanz, Germany). Where necessary, samples were re-sequenced to ensure comparable coverage across samples and read data from these technical replicates merged prior to alignment. 4 samples failed library preparation leaving a total of 74 samples. On average across the 74 samples we generated 50 +/- 11 (mean +/-SD) million reads per sample.

Sequence data quality control was evaluated using the FastQC program (http://www.bioinformatics.babraham.ac.uk/projects/fastqc/) and any reads with a substandard quality or adapter contamination were removed.

### Alignment and Differential expression analysis

Reads were aligned to the UCSC hg19 human reference genome using Tophat2 v2.0.3[68]. Up to two mismatches were allowed per uniquely aligned read. Alignment metrics were then collected using the *PICARD CollectRNASeqMetrics* (http://broadinstitute.github.io/picard/). On average across the 74 samples; 93% (+/- 0.4) of reads were uniquely mapped with an average of 84% mapping to mRNA (57% and 27% mapping to coding and UTR regions respectively).

After alignment, the read counts for each gene were summarized using the program *Htseq-count*[69] from the HTseq package. Normalisation and differential expression between each group of cases and controls was evaluated using DESeq2[30] version 1.6.0, implemented in R 3.01.1. DESeq2 uses a negative binomial generalised linear model (GLM) to evaluate differential gene expression. For significance testing, DESeq2 uses a Wald test statistic following normalisation based on size factors and variance. We adjusted for gender, sequencing batch and age covariates in the analysis. A gene base mean cut off of 10 and Padj ≤ 0.05, and a log2 fold change of above 1 or below -1 was used as a thresholds to define differential expression. Venn diagrams of overlapping genes between each group were generated using Venny (http://bioinfogp.cnb.csic.es/tools/venny/index.html).

To evaluate the possibility that different levels of T cell subpopulations were influencing our gene expression results in our dataset we examined transcript expression between the anti-CD3/CD28 stimulated and unstimulated coeliac case cohort and controls for a subset of CD4+ Th1 *(TBX21)*, Th2 *(GATA3)*, Th17 *(RORC)* and Treg *(FOXP3)* gene markers. From the four gene markers analysed, only one showed a significant divergence in transcript expression between our cases and controls at a threshold of *P* >0.05, the Th1 cell gene marker *TBX21* following anti-CD3/C28 stimulation (L2FC = 1.01,P_adj_ = 0.021). It should be noted that *TBX21* may be under the control of *BACH2* since it shows increased expression in Bach2 knock-out mice, and BACH2 is down regulated across all conditions (unstimulated log2FC = -0.72, P_adj_ = 0.052).

The raw sequencing reads (FASTQ files) and sequence read counts mapped to UCSC hg19 for each of the 74 transcriptomes sequenced in this study have been deposited at Gene Expression Omnibus (GEO) accession GSE69549.

### Power calculations for RNA Sequencing

The Bioconductor tool *RNASeqPower[70]* was used to calculate the power of our sample size to observe significant changes in expression. We estimated this based on our sample size of 11 biological replicates in the control group and 15 in the Coeliac group at a range of read depths using a coefficient of variation of 0.4.

### Pathway analysis of differentially expressed genes

GOseq[34] was used to identify gene ontology terms and KEGG biological pathways that were significantly enriched in differentially expressed genes from the RNA Seq analysis. Only genes considered expressed subsequent to DESeq2’s independent filtering were used as background. GOseq specifically adjusts for the higher abundance of reads from long or highly expressed genes in RNA-seq experiments when assessing a gene list for over-representation of biological functions. The false discovery rate from multiple-hypothesis testing was accounted for with Benjamini—Hochberg correction. GO terms and KEGG pathways with adjusted P values less than 0.05 were considered significantly enriched by differential expressed genes. To evaluate modules identified in WGCNA we used the commercially available software MetaCore (http://www.genego.com/metacore.php) which conducts pathway analysis based on manually curated database of transcription factors, protein-protein interactions and signaling and metabolic pathways. Pathways with adjusted p value <0.05 were considered significantly enriched for module genes.

### Analysis of differential exon usage using DEXSeq

We used the biooconductor package DEXSeq[36] (v1.12) to identify differential use of exons between coeliacs and controls. For a given exon, DEXSeq considers the ratio of the number of reads mapping to this exon compared to the number of reads mapping to any other exon of the same gene in each sample. Normalisation and dispersion estimates for each exon are calculated similarly to DESeq2 and the ratio of counts across conditions is used to infer differential usage of exons using a generalized linear model. Data was prepared as outlined in the package vignette correcting for age, gender and sequencing batch in the GLM.

### Gene co-expression network analysis

The R package WGCNA[24] (weighted gene co expression network analysis) was used to identify co-expression modules in a dataset consisting of our anti-CD3/CD28 stimulated cases and controls. Counts transformed using the *rlg* transformation implemented in DESeq2[30] was used as input. To reduce background noise, genes with less than 10 read counts in each sample were removed, resulting in a dataset consisting of 10,787 genes.

A signed co-expression network was constructed using WGCNA using standard procedures[24]. The adjacency matrix was created by calculating the bi-weight mid-correlation coefficients between all genes raised to a β power of 20 (based on the scale-free topology criterion recommendations for datasets of this size). Next genes were clustered into network modules using the Topological Overlap Measure (TOM). The dissimilarity TOM (1-TOM) was then used as input for hierarchical clustering, and modules (clusters of highly interconnected genes) were detected as branches of the dendrogram using the *DynamicTreeCut* algorithm. Highly correlated module genes are represented and summarized by their first principal component (referred to as the module eigengene, or ME). The ME is used to define measures of module membership (MM) or connectivity (kME) the correlation between a gene expression value and the module eigengene). Module hub genes (highly connected genes) were identified by ranking genes by module membership. Pearson’s correlations were calculated between the module eigengene and disease status in order to find modules that were correlated with coeliac disease. We used the *in silico* tool Disease Association Protein-Protein Link Evaluator (DAPPLE)[40] to test if there was an excess of protein-protein interactions within co expression modules. Genes from each module with a MM > 0.7 were submitted to DAPPLE as seed nodes and the resulting direct and indirect (through other proteins) interaction networks were built based on data from the InWeb database of protein interaction using 1,000 permutations and an interacting binding degree cut-off of 2.

### Testing for enrichment for genetic associations

We extracted the Immunochip assocation p-values from the Trynka et al study comprising 12014 individuals with coeliac disese and 12228 controls for SNPs located within genes differentially expressed in our anti-CD3/CD28 stimulated samples (excluding genes located with the MHC locus and those reaching GWS). The Quantile-quantile (QQ) plot was generated using Stata v10. We used INRICH (interval-based enrichment analysis for genome-wide association studies)[39] to evaluate whether differentially expressed genes between coeliacs and controls were enriched for Coeliac Immunochip *P*-values[7]. INRICH implements a permutation-based algorithm to test for enrichment whilst correcting for possible biases induced by marker density, linkage disequilibrium (LD) structure, and gene size. We first summarized Coeliac Immunochip results using the “*—clump*" algorithm in PLINK [71, 72]. We used *P*-value cutoffs of p_1_<0.01 (significance threshold for index SNPs) and p_2_<0.05 (the secondary significance threshold for clumped SNPs) and an r^2^ threshold of 0.2. INRICH tests whether there is enrichment within these associated regions (intervals) for particular sets of targets (in this instance; genes differentially expressed in our dataset). We applied 10,000 permutations in our first round of INRICH enrichment analysis and 5,000 in the bootstrap replication round for correction of multiple testing.

### Gene Set Enrichment Analysis

We used gene set enrichment analysis (GSEA) [44]to quantify enrichment of genes identified as regulated by BACH2 amongst genes that were differentially regulated between cases and controls using all three conditions (antiCD3/CD28, PMA and UNS). We used the gene sets discovered by Roychoudhuri et al [27] and Vahedi et al [43]. We used only genes which we could identify in the human genome from the Roychoudhuri gene set, giving a total of 123 genes and 98 in the Vahedi gene set. We ran the GSEA against normalised gene counts for the entire transcriptome for each condition with 1000 permutations and used an FDR q-value of 0.05 as significant.

## Supporting Information

S1 Fig Power to detect differential expression using RNA seq. Power estimates generated using RNASeqPower at varying fold change (FC) cutoffs and transcript coverage depths based on a sample size of 11 v 15 and a coefficient of variation of 0.4.
(TIFF)

S2 Fig Enrichment of BACH2 regulated genes amongst DE genes in CD4+ T cells stimulated with anti-CD3/CD28. Gene Set Enrichment Analysis (GSEA) enrichment plot and heat map for the Vahedi et al gene set comprising BACH2 super enhancer regulated genes in CD4+ T cells compared to our differentially expressed genes following stimulation with anti-CD3/CD28.
(TIF)

S1 File Flow cytometry graphs demonstrating the purity of CD4+ T cells, CD8+ T cells and CD14+ monocytes in whole blood samples before and after CD4+ T cell enrichment. (PDF)

S1 Table Sample Information and patient characteristics for Coeliac individuals sequenced in this study. (XLSX)

S2 Table Genes differentially expressed (up/down regulated in coeliacs) between cases and controls in our anti-CD3/CD28 stimulated samples. (XLSX)

S3 Table Genes differentially expressed (up/down regulated in coeliacs) between cases and controls in our PMA stimulated samples. (XLSX)

S4 Table Pathways identified by GOSeq as significantly enriched for genes differentially expressed in our PMA stimulated samples. (XLSX)

S5 Table Genes differentially expressed (up/down regulated in coeliacs) between cases and controls in our unstimulated stimulated samples. (XLSX)

S6 Table Genes differentially expressed in our anti-CD3/CD28 stimulated samples that are regulated by BACH2. (XLSX)

S7 Table Module information from WGCNA indicating each genes module assignment, module membership (MM) and pvalue. Gene significance (GS) r2 and p values (based on the correlation of a gene expression profile with disease status) are also included.
(XLSX)

S8 Table GSEA comparison table listing Vahedi et al gene set comprising BACH2 super enhancer regulated genes in CD4 T cells compared to our differentially expressed genes following stimulation with anti-CD3/CD28. (XLSX)

S9 Table Genes differentially expressed between our coeliac samples and controls that have been genetically associated with other immune diseases. (XLSX)
