# Supplementary figures and images for: Transcriptome Analysis of CD4+ T Cells in Coeliac Disease Reveals Imprint of BACH2 and IFNγ Regulation
Source: PLoS One. 2015 Oct 7;10(10):e0140049. doi: 10.1371/journal.pone.0140049 (PMC4596691; doi:10.1371/journal.pone.0140049)

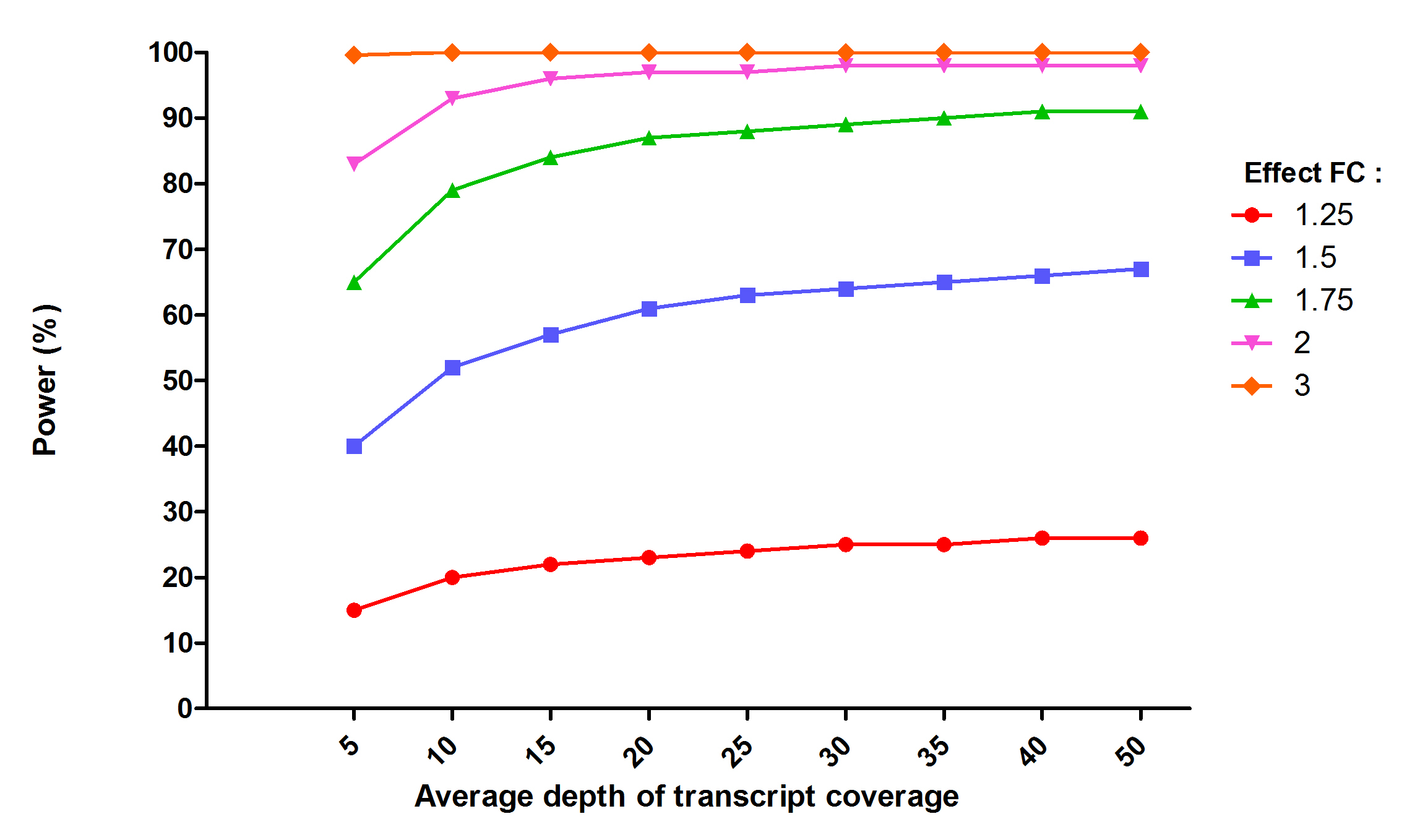

Supplement: S1 Fig — Power estimates generated using RNASeqPower at varying fold change (FC) cutoffs and transcript coverage depths based on a sample size of 11 v 15 and a coefficient of variation of 0.4. (TIFF) [file pone.0140049.s001.tiff]

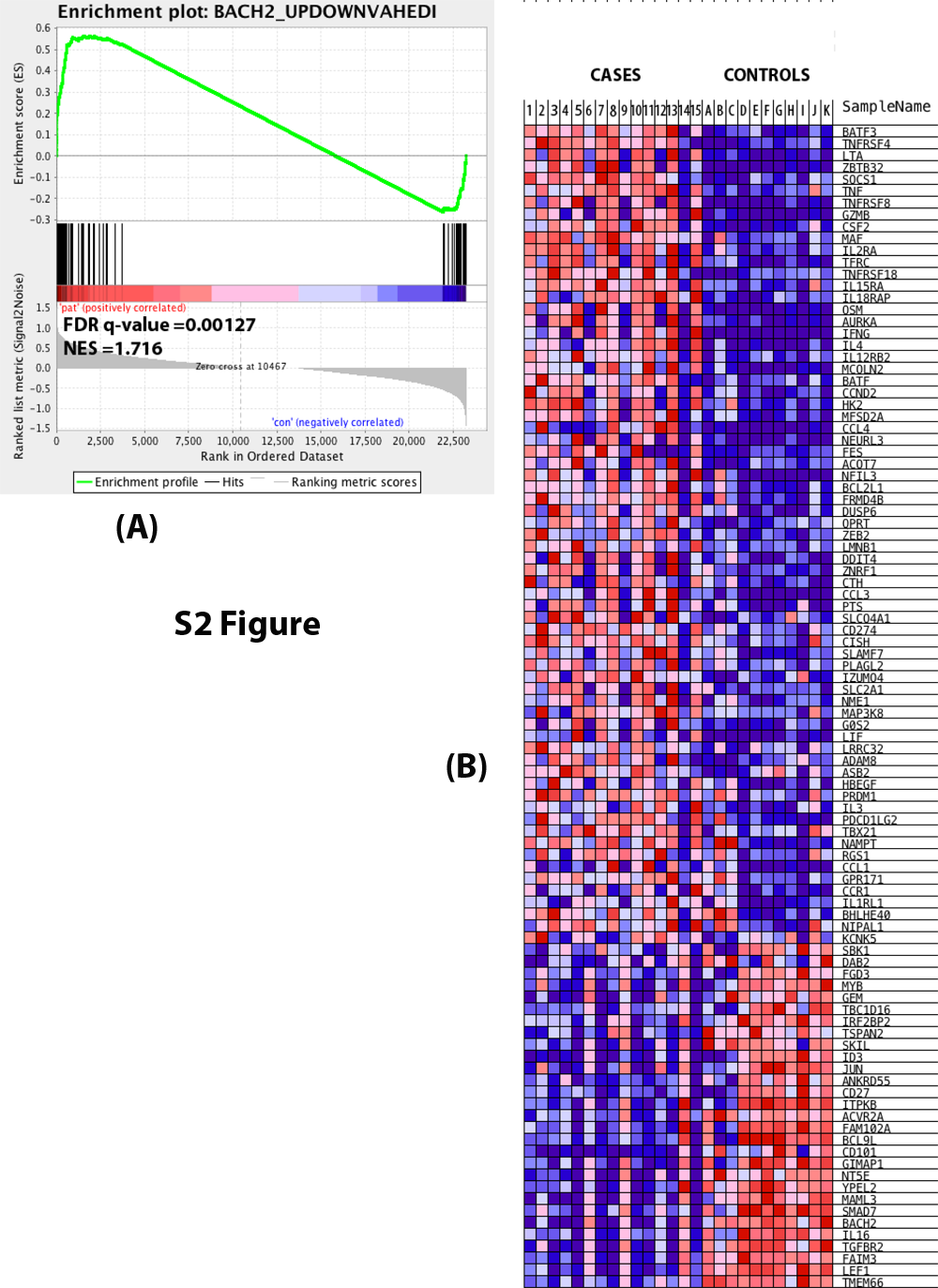

Supplement: S2 Fig — Gene Set Enrichment Analysis (GSEA) enrichment plot and heat map for the Vahedi et al gene set comprising BACH2 super enhancer regulated genes in CD4+ T cells compared to our differentially expressed genes following stimulation with anti-CD3/CD28. (TIF) [file pone.0140049.s002.tif]
