# Supplementary material for: Transcriptome Analysis of CD4+ T Cells in Coeliac Disease Reveals Imprint of BACH2 and IFNγ Regulation
Source: PLoS One. 2015 Oct 7;10(10):e0140049. doi: 10.1371/journal.pone.0140049 (PMC4596691; doi:10.1371/journal.pone.0140049)
Supplement: S1 File — (PDF) [file pone.0140049.s003.pdf]

Supplementary material

Graphs A, B and C show the purity of CD4+ T cells, CD8+ T cells and CD14+ monocytes in whole blood samples before and after CD4+ T cell enrichment.

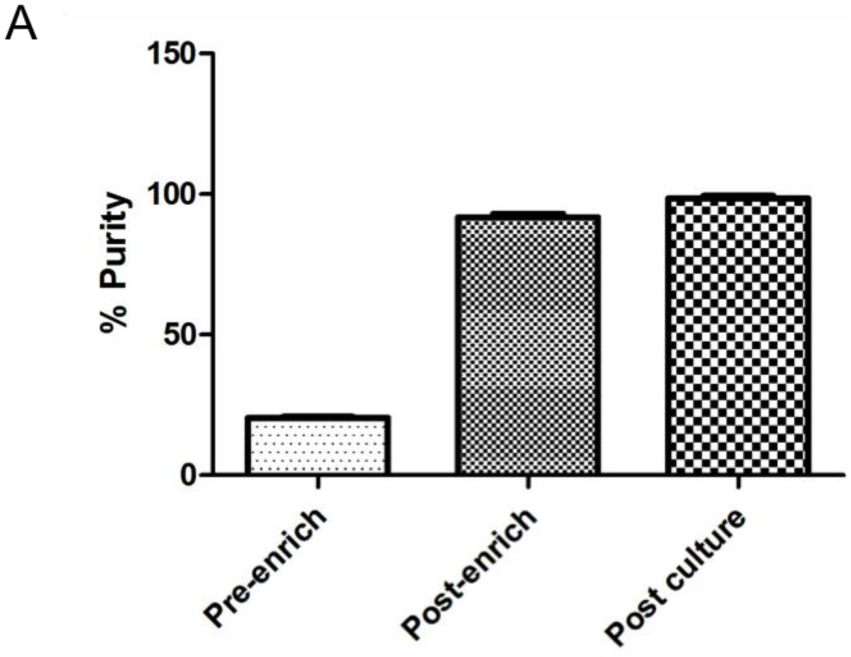

Figure A: Purity of CD4⁺ T cells from human peripheral blood leucocytes (20.52% pre –enrich), following enrichment for CD4⁺ T cells using CD4⁺ microbeads (92.01% post-enrich) and after incubation for 24 hours at 37° C (98.72% post culture).

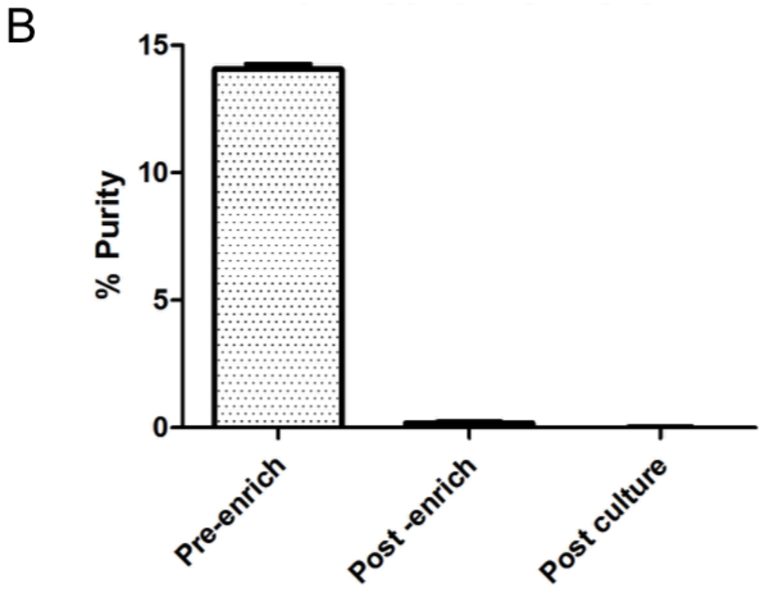

Figure B: Purity of $CD8^+$ T cells from human peripheral blood leucocytes (14.12% pre –enrich), following enrichment for $CD4^+$ T cells using $CD4^+$ microbeads (0.18% post-enrich) and after incubation for 24 hours at 37° C (0.01% post culture).

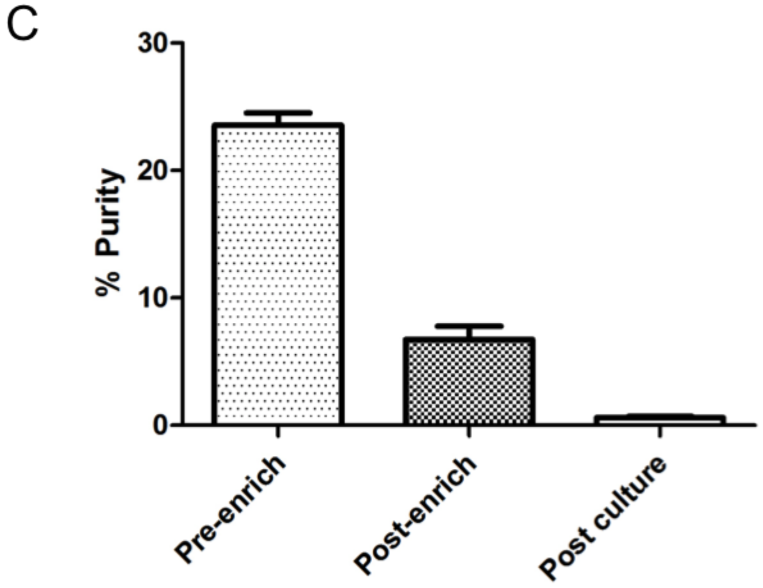

Figure C: Purity of $CD14^+$ T cells from human peripheral blood leucocytes (23.32% pre –enrich), following enrichment for $CD4^+$ T cells using $CD4^+$ microbeads (6.85% post-enrich) and after incubation for 24 hours at 37° C (0.62% post culture).

Flow cytometry graphs demonstrating the purity of CD4+ T cells, CD8+ T cells and CD14+monocytes in whole blood samples before and after CD4+ T cell enrichment. Replication 1 and 2 represent the first and second flow cytometric analyses respectively.

Replication 1

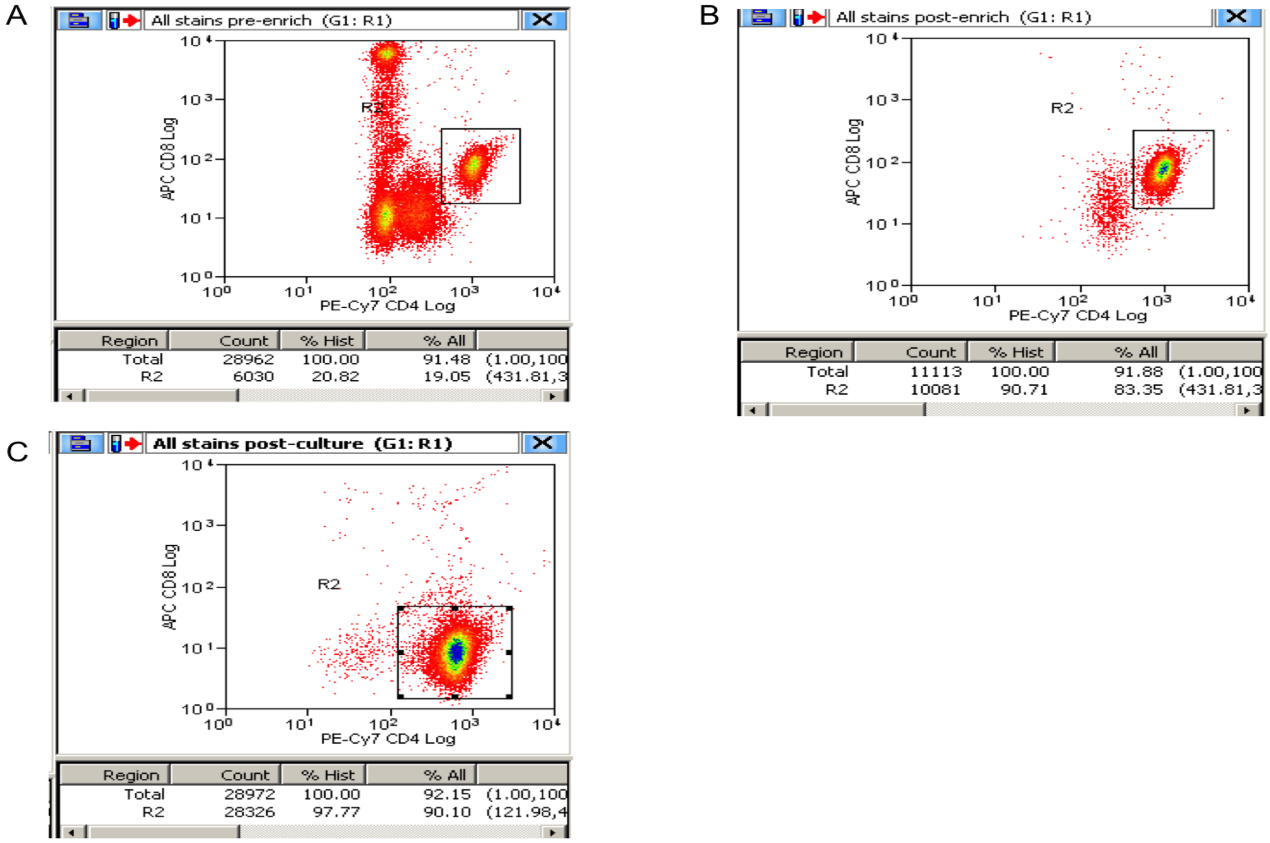

Figure D: Flow Cytometry analysis of $CD4^+$ T cells from human whole blood. $CD4^+$ T cells were identified using the display of PE-Cy7-CD4 (X-axis) vs APC-CD8 (Y-axis). The square (R2) highlights the population of T cells expressing the CD4 marker and negative for the CD8 marker in whole blood (A-pre-enrich), following enrichment for $CD4^+$ T cells (B-post-enrich) and after incubation for 24 hours at 37° C (C-post culture).

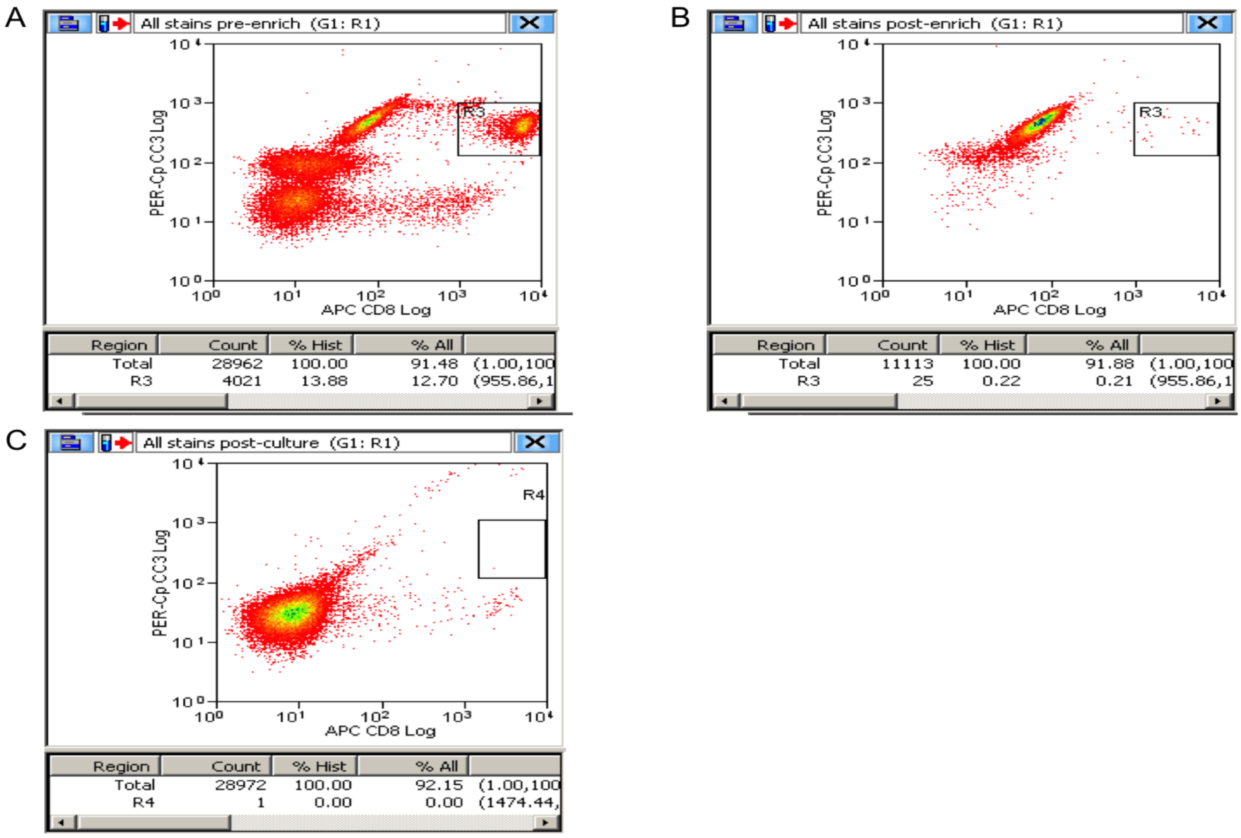

Figure E: Flow Cytometry analysis of CD8+ T cells from human whole blood. CD8+ T cells were identified using the display of APC-CD8 (X-axis) vs Per-CP-CD3 (Y-axis). The square (R3) highlights the population of T cells expressing both the CD8 and CD3 markers in whole blood (A-pre-enrich), following enrichment for CD4+ T cells (B-post-enrich) and R4 highlights the population of T cells expressing both the CD8 and CD3 markers after incubation for 24 hours at 37° C (C-post culture).

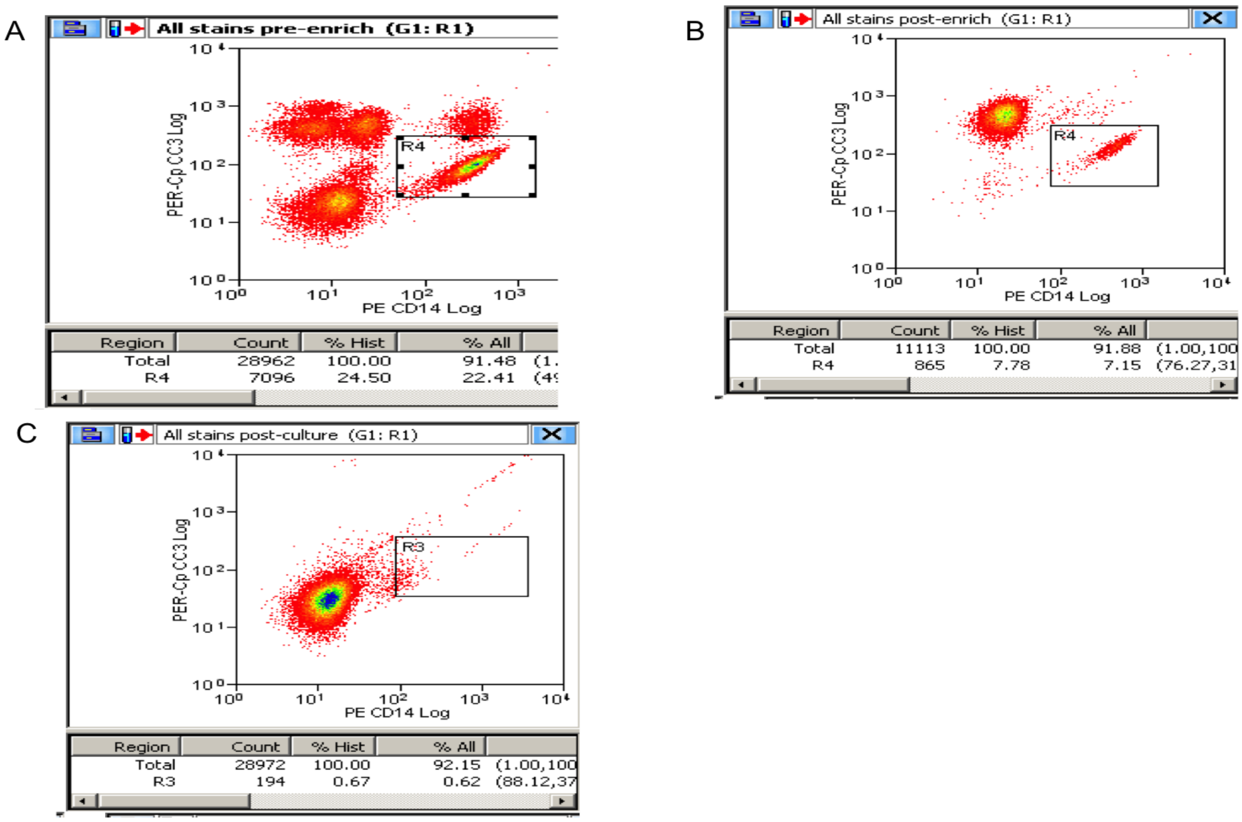

Figure F: Flow Cytometry analysis of $CD14^+$ T cells from human whole blood. $CD14^+$ T cells were identified using the display of PE-CD14 (X-axis) vs Per-CP-CD3 (Y-axis). The square (R4) highlights the population of T cells expressing the CD14 monocyte marker in whole blood (A-pre-enrich), following enrichment for $CD4^+$ T cells (B-post-enrich) and R3 highlights the population of T cells expressing the CD14 monocyte marker after incubation for 24 hours at 37° C (C-post culture).

.

Replication 2

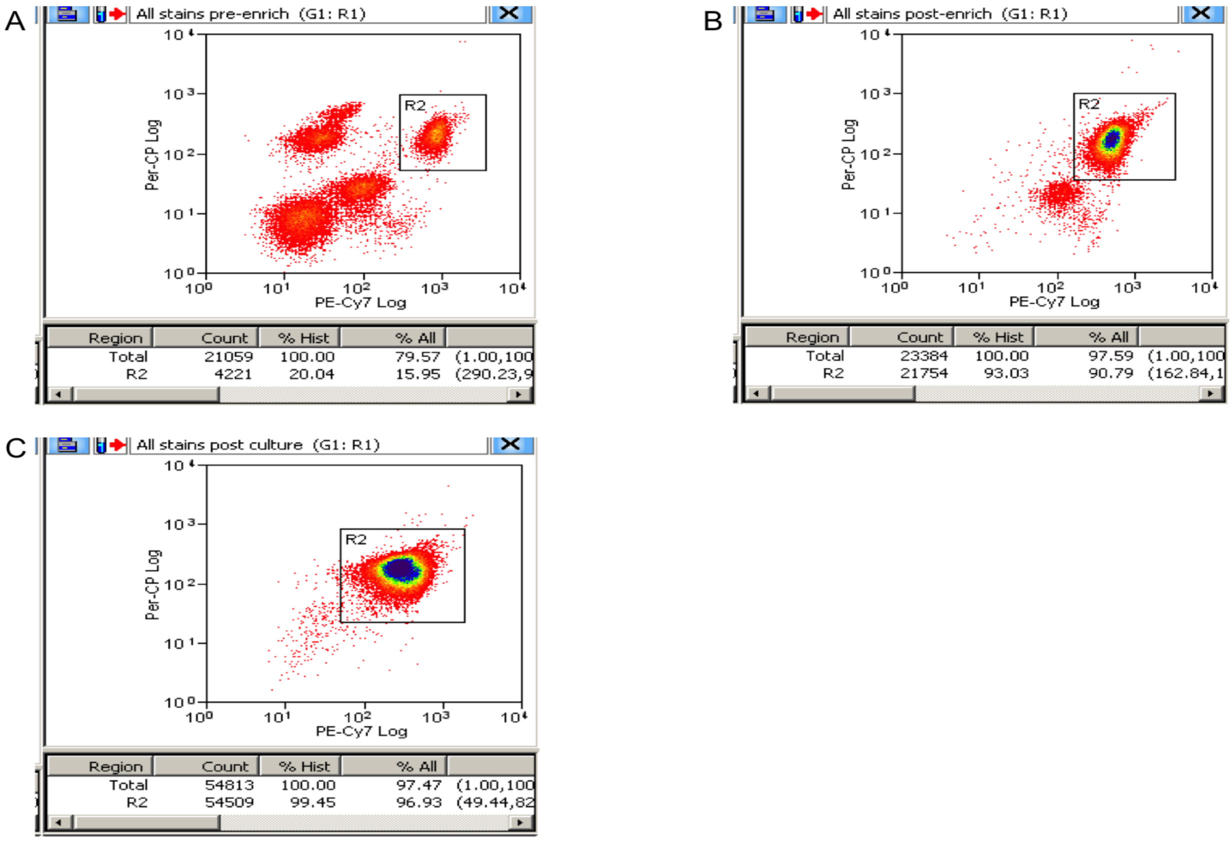

Figure G: Flow Cytometry analysis of $CD4^+$ T cells from human whole blood. $CD4^+$ T cells were identified using the display of PE-Cy7-CD4 (X-axis) vs PerCP-CD8 (Y-axis). The square (R2) highlights the population of T cells expressing the CD4 marker and negative for the CD8 marker in whole blood (A-pre-enrich), following enrichment for $CD4^+$ T cells (B-post-enrich) and after incubation for 24 hours at 37° C (C-post culture).

.

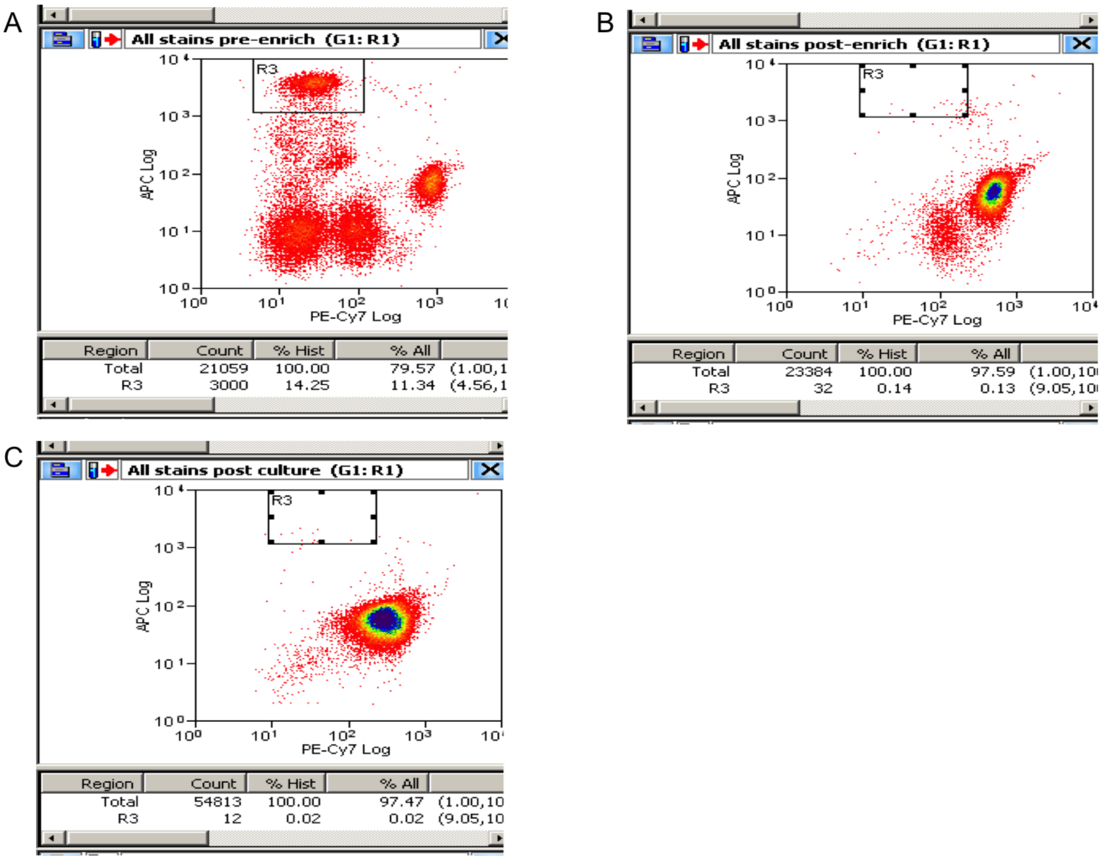

Figure H: Flow Cytometry analysis of $CD8^+$ T cells from human whole blood. $CD8^+$ T cells were identified using the display of PE-Cy7-CD4 (X-axis) vs APC-CD8 (Y-axis). The square (R3) highlights the population of T cells expressing the CD8 marker in whole blood (A-pre-enrich), following enrichment for $CD4^+$ T cells (B-post-enrich) and after incubation for 24 hours at 37° C (C-post culture).

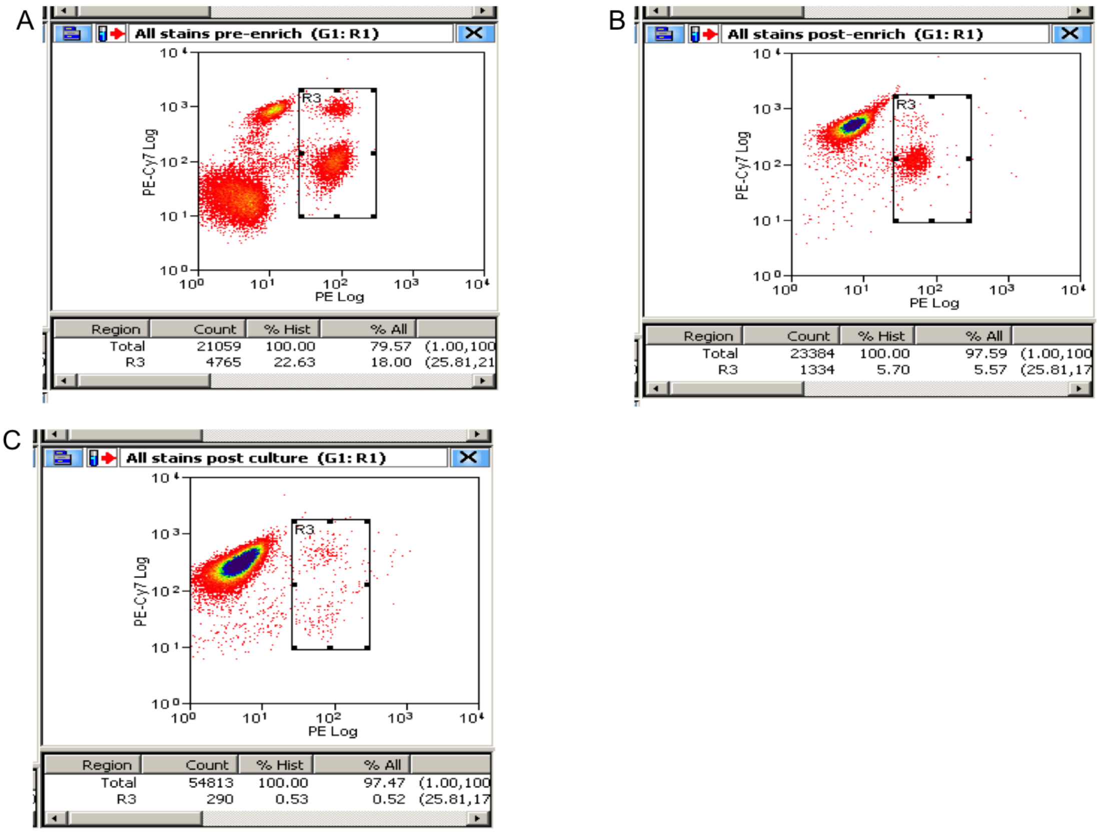

Figure I: Flow Cytometry analysis of $CD14^+$ T cells from human whole blood. $CD14^+$ T cells were identified using the display of PE-CD14 (X-axis) vs PE-Cy7 - CD3 (Y-axis). The square (R3) highlights the population of T cells expressing the CD14 monocyte marker in whole blood (A-pre-enrich), following enrichment for $CD4^+$ T cells (B-post-enrich) and after incubation for 24 hours at 37° C (C-post culture).
